# A Renal Olfactory Receptor Aids in Kidney Glucose Handling

**DOI:** 10.1038/srep35215

**Published:** 2016-10-14

**Authors:** Blythe D. Shepard, Lydie Cheval, Zita Peterlin, Stuart Firestein, Hermann Koepsell, Alain Doucet, Jennifer L. Pluznick

**Affiliations:** 1 Department of Physiology, Johns Hopkins University School of Medicine, Baltimore, MD 21205, USA; 2 Sorbonne Universités, UPMC Univ Paris 06, INSERM, Université Paris Descartes, Sorbonne Paris Cité, UMR_S 1138, CNRS, ERL 8228, Centre de Recherche des Cordeliers, Paris, France; 3 Department of Biological Sciences, Columbia University, New York, NY 10027, USA; 4 Department of Molecular Plant Physiology and Biophysics, Julius-von-Sachs-Institute, University Wurzburg, Julius-von-Sachs-Platz 2, 97082 Wurzburg, Germany

## Abstract

Olfactory receptors (ORs) are G protein-coupled receptors which serve important sensory functions beyond their role as odorant detectors in the olfactory epithelium. Here we describe a novel role for one of these ORs, Olfr1393, as a regulator of renal glucose handling. Olfr1393 is specifically expressed in the kidney proximal tubule, which is the site of renal glucose reabsorption. Olfr1393 knockout mice exhibit urinary glucose wasting and improved glucose tolerance, despite euglycemia and normal insulin levels. Consistent with this phenotype, Olfr1393 knockout mice have a significant decrease in luminal expression of Sglt1, a key renal glucose transporter, uncovering a novel regulatory pathway involving Olfr1393 and Sglt1. In addition, by utilizing a large scale screen of over 1400 chemicals we reveal the ligand profile of Olfr1393 for the first time, offering new insight into potential pathways of physiological regulation for this novel signaling pathway.

Olfactory receptors (ORs) are seven transmembrane domain G protein-coupled receptors (GPCRs) that serve as the chemical sensors of smell in the olfactory epithelium (OE). While these receptors were originally thought to be restricted to the nose^1^, it is now appreciated that ORs and other sensory receptors are found in a variety of other tissues where they play important physiological functions^2^,^3^,^4^,^5^,^6^,^7^,^8^,^9^. We previously reported that at least 10 different ORs as well as their downstream signaling components (adenylate cyclase 3 and the olfactory G protein) are expressed in the kidney, and that one of these renal ORs contributes to blood pressure regulation^6^,^10^. However, the functions of the remaining renal ORs have remained a mystery. In this study, we report for the first time the localization, ligand profile and physiological relevance of renal Olfactory Receptor 1393 (Olfr1393).

We find that Olfr1393 localizes to all three segments of the renal proximal tubule, which is the site of renal glucose reabsorption. Typically, an individual’s entire blood volume is filtered ~50 times/day, and because glucose is neither protein-bound nor complexed with macromolecules, it is freely filtered by the glomerulus^11^,^12^. Under normal conditions, the proximal tubule reabsorbs the entirety of the ~180 grams of glucose filtered per day from the ultra-filtrate, such that no glucose is detected in the final urine. Glucose reabsorption in the proximal tubule is mediated by two apical sodium-glucose co-transporters: Sglt2 (SCL5A2) and Sglt1 (SLC5A1)^11^,^12^,^13^,^14^. The low affinity, high-flux transporter, Sglt2, is localized to the apical membrane of the early proximal tubule (S1 and S2) and handles >90% of all glucose reabsorption. The remaining glucose is cleared from the lumen by the high affinity, low-flux transporter, Sglt1, in the straight proximal tubule (S3). While these transporters have been extensively characterized and explored as potential drug targets for type II diabetes^11^,^15^,^16^, the understanding of their regulation within the proximal tubule is limited^17^.

Here, we report that Olfr1393 knockout (KO) mice present with euglycemic glycosuria and improved glucose tolerance. Consistent with this, we observe an altered distribution of Sglt1 in the proximal tubule of KO animals, implicating Olfr1393 as a novel contributor to renal glucose handling. Additionally, when we began these studies, Olfr1393 was an ‘orphan’ receptor with no known ligands; therefore, we undertook a ligand screen and identified 8 ligands for Olfr1393. In sum, these studies have uncovered a novel role for a renal OR in kidney glucose handling, and have identified a novel mechanism for potential physiologic regulation of Sglt1.

## Results

### Olfr1393 is localized to the renal proximal tubule

**Figure 1:**
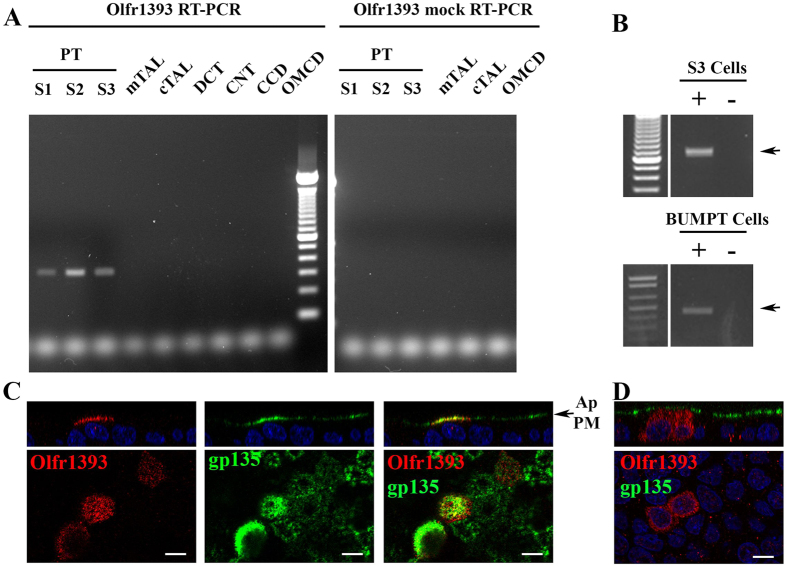
Olfr1393 is found in the renal proximal tubule. (**A**) Renal segments were micro-dissected and reverse-transcribed with (RT-PCR) or without (mock RT-PCR) the reverse transcriptase enzyme. A representative gel from 1 of 3 separate mice is shown, and all bands were sequenced to confirm their identity. In all cases, Olfr1393 is found in all three regions of the proximal tubule (PT): S1, S2 and S3. (TAL, thick ascending limb (m, medullary, or c, cortical); DCT, distal convoluted tubule; CNT, connecting tubule; CCD, cortical collecting duct; OMCD, outer medulary collecting duct). For segments where sufficient amounts were collected, mock RT-PCR reactions were also performed. Olfr1393 was not detected in any of the mock reactions including all three regions of the proximal tubule. (**B**) RT-PCR for Olfr1393 was performed on mouse S3 cells (top) and BUMPT cells (bottom) to detect endogenous expression. Olfr1393 was detected in both cell lines in the RT-PCR (+) but not in the mock RT-PCR reactions (−). (**C,D**) Polarized MDCK cells were transiently transfected with flag-tagged Olfr1393. To detect Olfr1393, cells were stained with an anti-flag antibody either by (**C**) surface labeling the apical membrane in live, non-permeabilized cells or (**D**) staining for total Olfr1393 in fixed and permeabilized cells. In both cases, cells were also stained for gp135 (green) to mark the apical membrane. In (**C**) the merged image indicates colocalization between Olfr1393 and gp135 at the apical plasma membrane (Ap PM). All scale bars indicate 10 μM.

Previously, we reported that Olfr1393 is expressed in whole murine kidney^6^ by RT-PCR. To further localize Olfr1393, individual nephron segments were dissected from whole kidney^18^,^19^,^20^ and nephron-segment RNA was isolated and reverse transcribed (RT; or, subjected to “mock” RT when available). Whereas RT-PCR for β-actin was positive for every segment tested (Supplemental Fig. 1), Olfr1393 was found in all three segments of the proximal tubule (S1, S2 and S3; Fig. 1A). Proximal tubule localization was observed in nephron segments isolated from three different mice, and importantly, the mock reactions were clean for all segments analyzed, including the proximal tubule (Supplemental Fig. 1). Olfr1393 was additionally detected in the distal convoluted tubule from one set of nephron segments, but it was absent in the other two (Supplemental Fig. 1). In all cases, Olfr1393 bands were sequenced to confirm identity. To further confirm proximal tubule localization, we screened mouse proximal tubule cell lines for Olfr1393. RT-PCR for Olfr1393 revealed that both murine S3^21^ and BUMPT cells^22^ have *endogenous* Olfr1393 expression (Fig. 1B) indicating that renal proximal tubule Olfr1393 expression is maintained *in vitro*.

To determine additional sites of Olfr1393 expression, we undertook an RT-PCR survey of other tissues (n = 3–8 samples for each tissue). While Olfr1393 is consistently found in whole kidney, distribution amongst other tissues was varied. We regularly detected Olfr1393 in whole brain, and found sporadic expression in tissues including the thymus, heart, small intestine, adipose, liver, lung and spleen (Supplemental Table 1). Regardless of Olfr1393 expression, all tissues screened were positive for β-actin, and all Olfr1393 bands were sequenced to confirm identity.

In the nose, ORs localize to the primary cilia on the apical plasma membrane (PM) of olfactory sensory neurons (OSNs). The epithelial cells lining the proximal tubule are also polarized, and have a single primary cilium and densely packed microvilli on the apical PM^23^,^24^; thus, we predicted that Olfr1393 is also expressed on the apical PM in polarized epithelial cells. Because we have been unable to identify a reliable antibody for Olfr1393, we planned to localize Olfr1393 by overexpressing flag-tagged Olfr1393 in polarized epithelial cells. However, we found that both S3 and BUMPT cells have low transfection efficiencies and are poor expressers of exogenously expressed flag-tagged Olfr1393. This is not at all uncommon; functional expression of exogenous ORs is quite difficult as they are often retained in the endoplasmic reticulum and degraded instead of being functionally expressed on the plasma membrane^8^,^25^. However, in screening additional renal epithelial cells lines we determined that polarized MDCK cells successfully traffic a subset of ORs, including Olfr1393. Thus, we used MDCK cells to determine subcellular localization. For these experiments, MDCK cells were grown on filters, transfected with flag-Olfr1393, and allowed to fully polarize. The apical PM was selectively labeled with a flag antibody in live, nonpermeabilized cells (Fig. 1C; red) and cells were subsequently fixed, permeabilized and stained for gp135, an endogenous apical protein^26^ (Fig. 1C; green). Both Olfr1393 and gp135 were found on the apical PM, and partial co-localization can be seen in the Z plane (Fig. 1C). MDCK cells expressing flag-Olfr1393 were also probed for total flag staining in fixed and permeabilized cells. Olfr1393 was never seen on the basolateral membrane (Fig. 1D) indicating that Olfr1393 traffics exclusively to the apical PM in polarized epithelial cells. When these cells were double-labeled with acetylated α-tubulin to mark primary cilia^27^, we did not observe colocalization - suggesting that unlike OSNs, renal Olfr1393 is apical but not ciliary.

To understand the function of Olfr1393 in the renal proximal tubule, we generated a whole animal KO in which the entire single exon encoding Olfr1393 was replaced by a LacZ reporter (Supplemental Fig. 2A). Both the KO and heterozygous mice are fertile and viable, and born in normal Mendelian ratios. As expected, we detected robust β-galactosidase expression in individual neurons distributed throughout zones II-IV of the OE^28^ in KO (but never wild type; WT) mice (Supplemental Fig. 3A,B). We also observed β-galactosidase expression in neurons in the vomeronasal organ (VNO; Supplemental Fig. 3C). However, we were unable to detect β-galactosidase outside of the VNO or OE. Individual OSNs express high copy numbers of each OR^29^; in contrast, the low expression of ORs in the kidney and elsewhere likely prevents useful detection (compare expression differences between Olfr1393 and β-actin RT-PCR in Supplemental Fig. 1B). In support of this hypothesis, we screened WT and KO whole kidneys for LacZ and Olfr1393 by RT-PCR (Supplemental Fig. 2C). As expected, LacZ *was* detected in the KO kidney, while Olfr1393 was only found in the WT kidney.

### Olfr1393 KO mice are glycosuric with increased glucose tolerance

**Figure 2:**
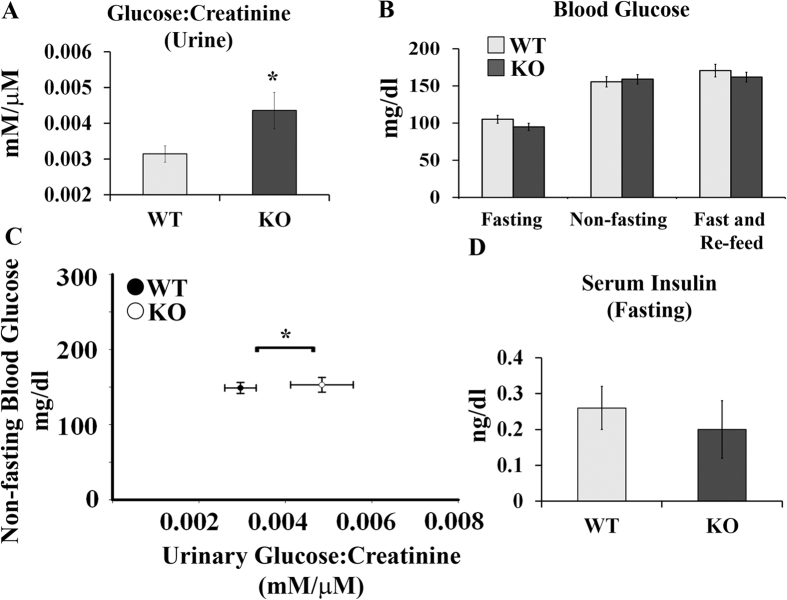
Olfr1393 KO mice present with euglycemic glycosuria. (**A**) KO mice have an increase in the urinary glucose:creatinine ratio. (WT n = 31 [14 males, 17 females], KO n = 28 [14 males, 14 females]). (**B**) Blood was collected from WT and KO mice that were either fasted overnight (WT n = 29 [16 males, 13 females], KO n = 25 [13 males, 12 females]), non-fasted (WT n = 21 [14 males, 7 females], KO n = 25 [11 males, 14 females]) or fasted overnight followed by a 2 hour period of re-feeding (WT n = 16 [5 males, 11 females], KO n = 11 [7 males, 4 females]). Blood glucose was measured by glucometer and no differences were detected between WT and KO mice. (**C**) Blood glucose (by glucometer) and urinary glucose:creatinine were measured in a subset of mice at the same time (WT n = 17 [10 males, 7 females], KO n = 15 [6 males, 9 females]). As was observed in A and B, KO mice present with an increase in urinary glucose:creatinine despite no differences in blood glucose. (**D**) Mice were fasted overnight and whole blood was collected and spun to isolate the serum. Insulin was measured by ELISA in WT and KO mice with no differences detected between genotypes (WT n = 6 [4 males, 2 females], KO n = 6 [4 males, 2 females]). Data represent ± SEM and * indicates p ≤ 0.05 by the student t-test.

**Figure 3:**
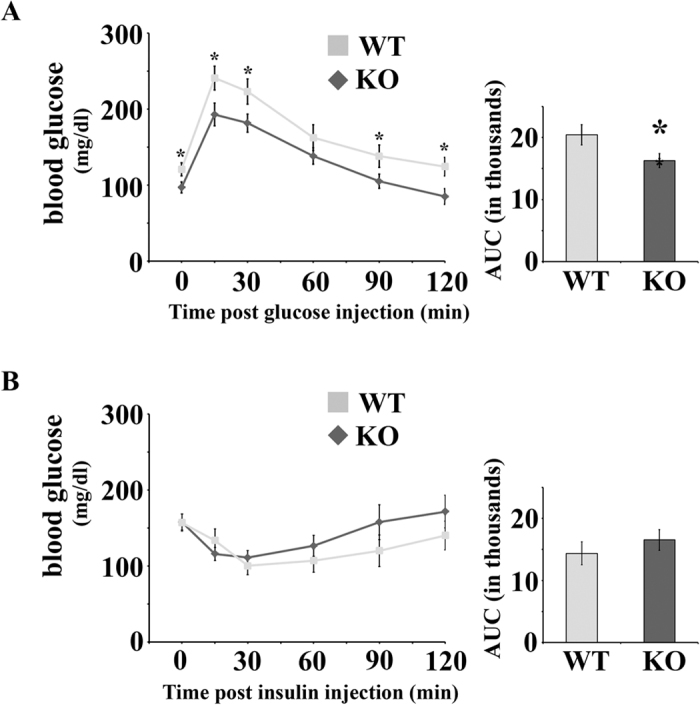
Olfr1393 KO mice have improved glucose tolerance as measured by GTT, but perform similarly on an ITT. (**A**) To perform a GTT, WT and KO mice were fasted overnight and injected with 1 g glucose/kg BW. Blood glucose was measured at 0, 15, 30, 60, 90 and 120 minutes following glucose injection. The area under the curve (AUC; in thousands) is displayed to the right of the GTT curve. (WT n = 12 [8 males, 4 females], KO n = 11 [6 males, 5 females]). (**B**) To perform an ITT, WT and KO mice were fasted for 2 hours and then injected with 0.7 U of insulin. Blood glucose was measured at 0, 15, 30, 60, 90 and 120 minutes following insulin injection and the AUC (in thousands) is displayed to the right of the ITT curve. (WT n = 8 [4 males, 4 females], KO n = 9 [5 males, 4 females]) Data represent ± SEM and * indicates p ≤ 0.05 by the student t-test.

As seen in Table 1, initial phenotyping of the KO mice indicated that these mice are similar to their WT littermates with respect to body weight, kidney weight, plasma electrolytes, systolic blood pressure (measured by tail cuff) and glomerular filtration rate (GFR; measured by transcutaneous decay of FITC-sinistrin). However, when random spot urines were analyzed by dipstick, a subset of KO mice were positive for urinary glucose. Subsequently, we measured the urinary glucose-creatinine ratio and found that Olfr1393 KO mice present with a mild, but significant glycosuria (Fig. 2A). Typically, urinary glucose wasting is only observed when blood glucose levels are high, and thus the amount of filtered glucose exceeds the maximum reabsorptive capacity of the proximal tubule. However, the fasted, non-fasted, and fast/re-feed blood glucose values from the KO mice were not different than the values detected for WT littermates (Fig. 2B). To rule out a transient hyperglycemia, we also measured non-fasting blood glucose levels at the same time as urine collection for a subset of mice. As seen in Fig. 2C, the urinary glucose-creatinine levels were significantly elevated in this group of KO mice despite no differences in their blood glucose. Therefore, we conclude that Olfr1393 KO mice exhibit euglycemic glycosuria, indicative of a proximal tubule defect^11^,^12^. In support of this, KO mice did not have elevated protein/creatinine ratios, suggesting a *specific* deficit in glucose handling (rather than general proximal tubule impairment). Fasting serum insulin values were also indistinguishable between WT and KO mice (Fig. 2D). Given their mild glucose wasting, we tested the ability of WT and KO mice to handle an influx of glucose using a glucose tolerance test (GTT). In WT mice, injection of glucose led to a rapid spike in blood glucose levels and a return to baseline by 120 mins (Fig. 3A). However, in KO mice, the peak blood glucose level was significantly lower, and returned to baseline earlier (Fig. 3A). Although the initial fasting glucose (time 0) was also slightly but significantly lower in KO mice in this GTT cohort, this finding did not hold true in the context of a larger n (see Fig. 2B). Despite the improvement in glucose tolerance, both the WT and KO mice performed similarly when challenged with insulin during an insulin tolerance test (ITT, Fig. 3B). In this case, administration of insulin led to a rapid decrease in blood glucose followed by a swift recovery. Taken together, this data suggests that the Olfr1393 KO mice have improved glucose tolerance that likely stems from their mild urinary glucose wasting, rather than altered metabolism.

### Olfr1393 and sodium glucose co-transporters

**Figure 4:**
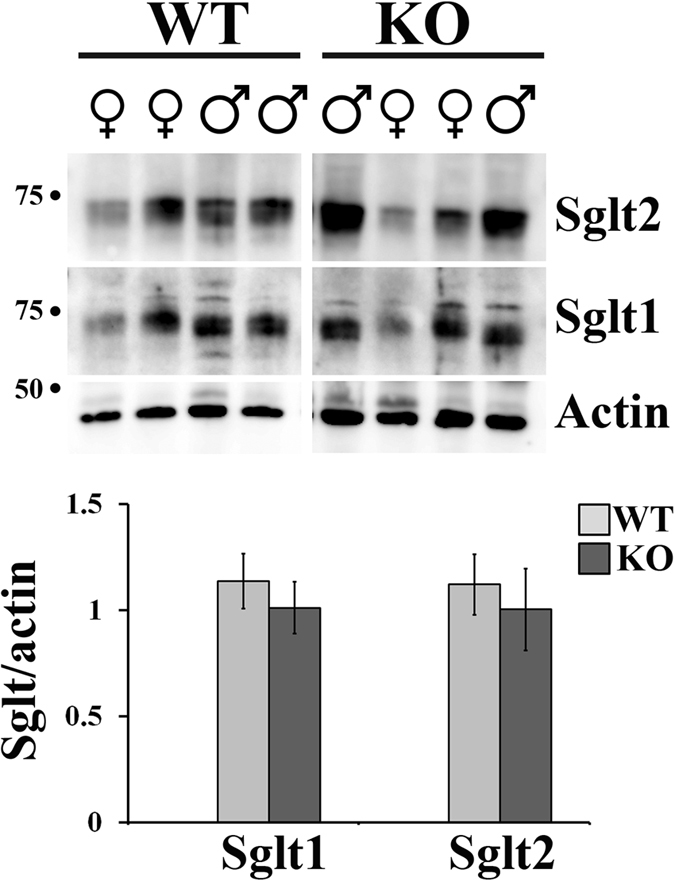
Total cellular membrane expression of Sglt1 and Sglt2 is unchanged between Olfr1393 WT and KO mice. Total cellular membranes were isolated from whole kidneys from both male and female WT and KO mice and immunoblotted for Sglt1 or Sglt2. (WT n = 8 [4 males, 4 females], KO n = 7 [4 males, 3 females]). Densitometry using ImageJ software confirmed that there are no differences in total expression when normalized to actin levels (graph). Data represent mean ± SEM.

**Figure 5:**
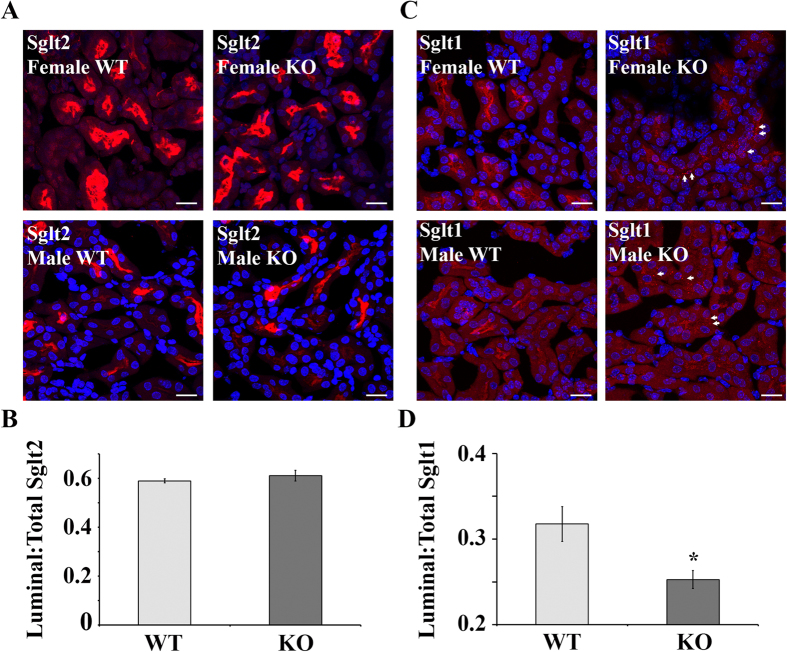
Kidneys from Olfr1393 KO mice have altered Sglt1 distribution. Kidneys from WT and KO mice were perfused and stained for Sglt2 (**A**) and Sglt1 (**C**). Representative images from both male and female littermates are shown, and in all cases, Sglt2 staining was unchanged, and Sglt1 luminal staining was reduced in Olfr1393 KO mice. For Sglt1, the arrows indicate regions of intracellular, punctate-like accumulation. The scale bar = 20 μM. (**B,D**) To quantify the luminal distribution of Sglt1 and Sglt2, the mean fluorescence intensity of the luminal and total tubule stain was measured for each LTL + tubule expressing Sglt. The ratio of the luminal to total stain was calculated. In (**B**) WT: 0.59 ± 0.008 (n = 3 kidneys [1 male, 2 female]), KO: 0.61 ± 0.02 (n = 5 kidneys [4 male, 1 female]). In (**D**) WT: 0.32 ± 0.02 (n = 4 kidneys [2 male, 2 female]), KO: 0.25 ± 0.01 (n = 6 kidneys [4 male, 2 female]). Data represent mean ± SEM and * indicates p ≤ 0.05 by the student t-test.

Glucose reabsorption is handled by two sodium-glucose co-transporters in the renal proximal tubule: Sglt2 (SCL5A2) and Sglt1 (SLC5A1)^11^,^12^,^13^,^14^. Interestingly, mice that are null for Sglt1 or Sglt2 also present with euglycemic glycosuria and improved glucose tolerance^13^,^14^,^30^. Given the similar phenotypes between Sglt1, Sglt2 and Olfr1393 KOs, and the common localization to the renal proximal tubule, we wondered if Olfr1393 may play a role in regulating Sglt1 and/or Sglt2 in the kidney. Western blotting revealed that the total cellular membrane expression of Sglt1 and Sglt2 is not altered in Olfr1393 KO mice (Fig. 4). However, despite the fact that there are no differences in total membrane expression, we noted that the intracellular localization of Sglt1 (as visualized by immunohistochemistry) is altered in Olfr1393 KO. For these experiments, kidney sections from WT and KO mice were stained with the fluorescein labeled lotus tetragonolobus lectin (LTL), to label proximal tubules and either Sglt2 (Fig. 5A) or Sglt1 (Fig. 5C). Both Sglt1 and Sglt2 were highly expressed along the proximal tubule lumen in WT mice (Fig. 5A,C), and this staining pattern remained unchanged for Sglt2 in the KO mice. However, we observed a diminished stain for Sglt1 near the luminal membrane in the KOs, with a corresponding increase in a punctate-like stain throughout the proximal tubules (Fig. 5C KO images, arrows). To quantify this observation, the ratio of luminal to total stain was determined for all LTL + tubules expressing Sglt1. As seen in Fig. 5D, the average luminal/total Sglt1 stain was 0.31 ± 0.002 for WT and 0.25 ± 0.01 for KO, representing a 22% decrease in the luminal stain (p = 0.01). Similar quantification of the Sglt2 images confirmed that Sglt2 localization is unchanged in Olfr1393 KO mice.

The altered distribution of Sglt1 in Olfr1393 KO mice suggests that Olfr1393 may promote apical localization of Sglt1, perhaps via a protein-protein interaction. To test whether Olfr1393 and Sglt1 can directly interact, HEK293T cells were transfected with Flag-Olfr1393, HA-Sglt1 or both constructs together and co-immunoprecipitation was performed using flag-conjugated agarose beads (Supplemental Fig. 4). When Olfr1393 was expressed, it was completely recovered in the bound (B) fraction. Although we observed a portion of Sglt1 in the unbound (UB) fraction when Olfr1393 and Sglt1 were co-expressed, Sglt1 did partially co-immunoprecipitate with Olfr1393.

### Olfr1393 ligands

Olfr1393, like the majority of olfactory receptors, is an orphan receptor with no known ligand. Therefore, we undertook a ligand screen using a cAMP-dependent luciferase reporter assay^31^. After confirming the functional surface expression of Olfr1393 in HEK293T cells (Supplemental Fig. 5), we performed an unbiased screen using different odorant mixtures, each containing a variety of chemicals clustered by functional group such that the mixes, in sum, cover a wide “odorant space”. The mixes used (MA, OxlK, BzC, Thi-Di) were tested as previously described^10^. However, we did not observe any Olfr1393 activation using these mixes.

**Figure 6:**
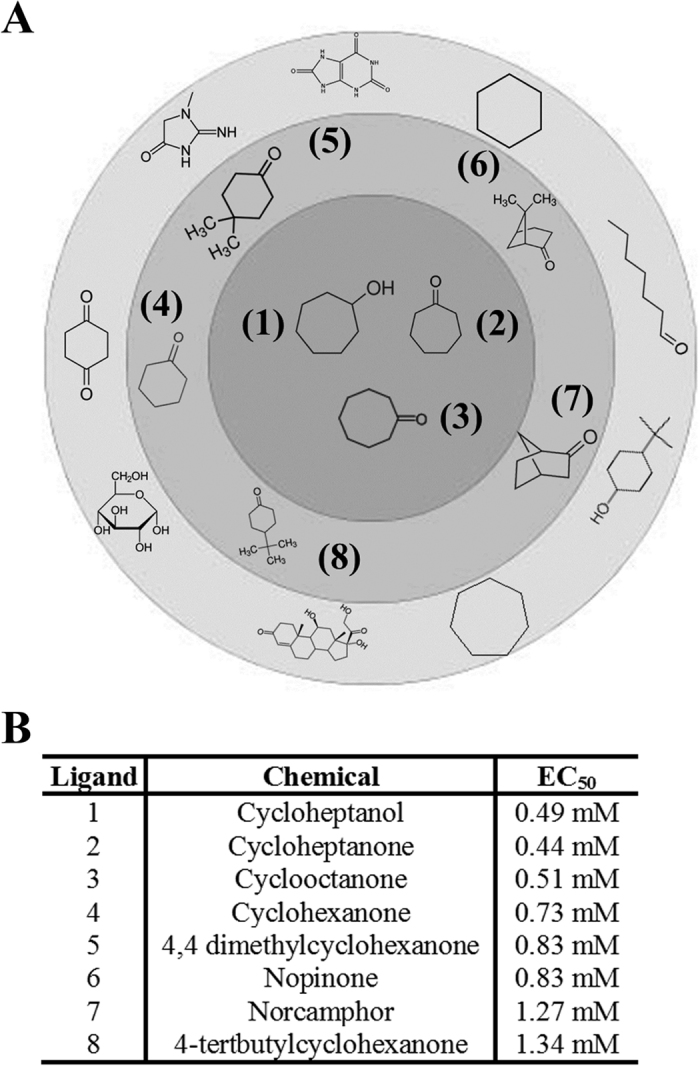
Olfr1393 responds to chemicals with pre-constrained rings containing either ketones or alcohols. (**A**) Chemical structures for the 8 identified ligands for Olfr1393 are represented in the inner two circles. The strongest ligands are found in the dark gray inner circle while the weaker ligands are found in the light gray middle ring. Examples of structurally similar chemicals that do not activate Olfr1393 are found in the outermost ring. A complete list of chemicals that did not activate Olfr1393 can be found in Supplemental Table 2. The activating chemicals are numbered 1–8 and correspond to the labeling in the table in (**B**).

The murine OR gene family (consisting of ~1000 ORs) is subdivided into subfamilies, grouped based on phylogenetic clustering and protein sequence identity^32^, and ORs within a subfamily often share similar ligands. Olfr1393 belongs to the MOR256 subfamily, and several other MOR256 family members have been previously deorphanized^33^,^34^,^35^,^36^,^37^. Thus, we screened Olfr1393 against known MOR256 odorants and found that Olfr1393 did respond to one of these chemicals – cyclohexanone (chemical #4 on Fig. 6). Using cyclohexanone as a guide, we broadened our search by using a multidimensional physiochemical metric for odorant prediction and identified a total of 8 different ligands^38^ (Fig. 6 and Supplemental Figs 6 and 7). As seen in Fig. 6A, these 8 ligands are all structurally similar, and are broadly classified as pre-constrained rings containing ketone or alcohol functional groups (middle two rings). Notably, modifying the ligands even slightly with the addition or removal of a functional group does not activate the receptor (Fig. 6A, outermost circle). An additional unbiased screen (using a 1280 compound chemical library) did not activate Olfr1393, indicating the narrow range of its ligand profile.

As there are ~1000 murine ORs and most remain orphan receptors, it is difficult to rule out the possibility that some of these 8 identified Olfr1393 ligands may activate other ORs. However, the most closely related OR to Olfr1393 by sequence similarity is Olfr1392, which differs from Olfr1393 by only 15 amino acids, and is also found in the kidney^6^. Therefore, to test for ligand specificity, we screened the Olfr1393 ligands on cells expressing Olfr1393, Olfr1392 or an empty vector, using the luciferase assay (Olfr1392 surface expression in HEK293T cells was confirmed; Supplemental Fig. 5). As seen in Supplemental Fig. 6, none of the Olfr1393 ligands activated Olfr1392, indicating that these ligands are relatively specific for Olfr1393.

## Discussion

We report here that a renal olfactory receptor – Olfr1393 – plays a physiological role in renal glucose handling. This is also the first report of identified ligands for Olfr1393. These findings expand the known roles of olfactory receptors in ‘ectopic’ tissues, and identify a physiological modulator of a clinically important pathway: Sglt-mediated glucose reabsorption.

Olfr1393 KO mice present with mild glycosuria and improved glucose tolerance. Despite this, their blood glucose and serum insulin values are normal. Typically, glycosuria is accompanied by hyperglycemia; in the absence of hyperglycemia, glycosuria indicates that the threshold for proximal tubule glucose reabsorption is lowered^11^,^12^. In keeping with this, kidneys from Olfr1393 KO mice have decreased luminal Sglt1 staining. Interestingly, the Olfr1393 phenotype is remarkably similar to what is described for both Sglt1 and Sglt2 KO mice, which present with euglycemic glycosuria, normal insulin levels, and improved glucose tolerance^13^,^14^,^30^. While mice and humans lacking Sglt2 excrete large quantities of glucose in the urine (~1800 μM/mg creatinine^14^), Sglt1 KO mice present with a milder phenotype. This aligns well with the understanding that Sglt2 handles >90% of glucose reabsorption, while Sglt1 reabsorbs the remaining 3–10%^11^,^12^,^13^,^14^. Thus, the relatively mild glycosuria seen in both Sglt1 and Olfr1393 KO mice is entirely consistent with our hypothesis, outlined below, that Olfr1393 serves as a modulator of Sglt1 function. It is also worth noting that urinary glucose wasting (induced via Sglt inhibition or genetic KO) has been used as a mechanism for lowering blood glucose levels. However, glucose lowering is not observed until blood glucose values are elevated above normal, as is the case in diabetic mouse models; that is, blood glucose values are not altered in Sglt1 or Sglt2 KOs on normal chow diets^14^,^39^. In future studies, it will be exciting to challenge glucose regulation in Olfr1393 KO mice to determine if their mild glucose wasting can similarly protect blood glucose values in a more ‘pathophysiological’ setting.

Because we observed a decrease in luminally-associated Sglt1 in KO mice despite similar total cellular membrane expression, Sglt1 may be either accumulating in intracellular vesicles or being actively retrieved from the apical PM for recycling or degradation. While little is known about the trafficking of Sglt1 (or Sglt2) within the proximal tubule epithelium, a role has been described for the RS1 protein in modulating dynamin-dependent release of Sglt1 from the Trans Golgi Network^40^,^41^,^42^. In addition, it has been shown that increased cAMP leads to an increase in Sglt1 on the plasma membrane^17^,^43^,^44^,^45^,^46^. In the canonical OR signaling pathway, OR activation leads to a rise in cAMP levels. Thus, the absence of Olfr1393 should result in lower cAMP levels, consistent with the decreased luminal stain that we observed. Clearly, further work is required to elucidate the full downstream signaling pathway for Olfr1393, to determine how the Sglt1 trafficking pathway is controlled by Olfr1393 signaling, and to determine what effect this altered localization has on Sglt1 (and Sglt2) activity. In addition, in the future it would be informative to quantitate total Sglt1 and Sglt2 levels using purified proximal tubule preparations, rather than whole kidney, to further ensure that total Sglt1 expression levels are unchanged in Olfr1393 KO mice.

Our data implies that Olfr1393’s role is to regulate the final step in renal glucose reabsorption: Sglt1. As a high-affinity transporter in the final segment of the proximal tubule, Sgtl1 plays a crucial role in ensuring that filtered glucose is completely recovered from the filtrate and is not lost in the urine. This concept is very much in keeping with a general ‘theme’ in renal transport: many substrates are reabsorbed in bulk early on, whereas fine-tune regulation happens in the latter segments of the nephron. For example, the great majority of filtered sodium is reabsorbed in the proximal tubule (~67%) and thick ascending limb (~25%). However, in a healthy individual sodium balance is determined by what happens in the distal nephron, where the final 8% of filtered sodium is handled^47^,^48^,^49^,^50^. Even a small adjustment in the handing of this filtered sodium (or glucose) can easily result in a significant change in excretion due to the fact that the entire blood volume is filtered many times per day. Thus, the observed 22% decrease in luminal Sglt1 may indeed be sufficient to cause (mild) glycosuria. In future studies, it will be important to identify and investigate physiologic (or pathophysiologic) circumstances under which the kidney chooses to ‘adjust’ Sglt1 function, and therefore, to perhaps permit small amounts of glycosuria.

In addition to altered Sglt1 localization, we also observed a direct interaction between Olfr1393 and Sglt1. While the co-immunoprecipitations were done *in vitro*, the interaction between these proteins supports the notion that Olfr1393 is contributing to the regulation of Sglt1. The immunoprecipitation was performed in the absence of an Olfr1393 ligand; thus, it is possible that the interaction between Olfr1393 and Sglt1 would be enhanced in the presence of its ligand.

Of note, all data presented include combined measurements taken from both males and females. Although we did not detect sex differences in the majority of these measurements, we did note an increase in Sglt2 total cellular membrane expression in male vs. female KOs (p = 0.04), concurrent with a lower rate of glycosuria in male vs female KOs (p = 0.03). While the change in Sglt1 localization occurred similarly in both male and female KOs, the increase in Sglt2 expression in male KOs may attenuate the glycosuria phenotype. It should be noted, however, that not all of our studies were powered to detect sex differences (i.e. fasting insulin in Fig. 2 and Sglt localization in Fig. 5) and in the future, it will be important to carefully examine any potential differences. Sex differences for Sglt1 and Sglt2 expression have been previously noted in both mice and rats^51^,^52^, and it will be interesting to determine whether the mechanism of Olfr1393 regulation of Sglt1 is also influenced by sex.

The phenotype of Olfr1393 KO mice (euglycemic glycosuria) is highly indicative of a renal proximal tubule defect. However, our studies have been performed with whole animal KO mice and we cannot rule out the possibility that their phenotype is influenced by the non-renal functions of Olfr1393. The generation of tissue-specific Olfr1393 KOs would help to clarify Olfr1393’s role in renal glucose handling and shed light on its other functions. As shown in Supplementary Table 1, we did find that Olfr1393 could be detected in a number of different tissues including the brain, heart and thymus. Our tissue expression screens were performed on tissues obtained from both male and female C57BL6 mice, and while there was mouse-to-mouse variability in our findings, none of these differences could be attributed to the age or sex of the animal. We suspect that the failure to detect Olfr1393 in all tissues of a given type (i.e., Olfr1393 was found in 4/5 brain samples) likely indicates that Olfr1393 expression is quite low and near the detection threshold for RT-PCR (although we cannot rule out true physiological variability amongst different animals). Despite this variability in detection, it is clear that Olfr1393 has weak to no expression in several tissues which play key roles in glucose regulation (rarely seen in adipose and never found in pancreas), supporting its renal-specific role in glucose handling. Of note, Olfr1393 was also rarely detected in the small intestine (2/7 samples), the other primary site of Sglt1 expression. In addition to glucose wasting, inhibition of Sglt1 in humans and mice leads to glucose/galactose malabsorption due to its intestinal localization^39^. This phenotype was not detected in our Olfr1393 KO mice suggesting that the Olfr1393-Sglt1 regulation is indeed renal specific, or alternatively, that Olfr1393 acts to fine-tune Sglt1 regulation but is not required for basal activity in the intestine.

Given the role for Olfr1393 in glucose handling and Sglt1 regulation, understanding the Olfr1393 signaling pathway is important. This study identified a total of 8 ligands for Olfr1393, all with similar chemical structures. To date, the physiological relevance of these ligands is still unknown; however, at least some of the Olfr1393 ligands are present physiologically: cyclohexanone has been found in the expelled breath and urine of patients with metastatic lung cancer, and in urine from patients with diabetes^53^,^54^. Therefore, it is likely that Olfr1393, positioned on the apical PM in the proximal tubule, could come in contact with cyclohexanone or a related chemical. However, none of these ligands activate Olfr1393 in the nanomolar range, the typical concentration range for OR activation, and it is not yet known whether plasma and urine cyclohexanone concentrations are high enough to activate Olfr1393 *in vivo* (cyclohexanone EC_50_ = 0.73 mM). To date, most metabolomics screens done in urine and plasma are untargeted and will only detect the most abundant chemicals – therefore, targeted metabolomics screens will be required to document the physiological levels of these compounds in urine and plasma. Conversely, although we cannot rule out that there may be additional Olfr1393 ligands which we have yet to identify, we did undertake an unbiased screen of a chemical library (~1400 chemicals in total) without identifying any additional ligands (Supplementary Table 2). In future studies, it will be crucial to explore the physiological role of Olfr1393 ligands in renal function.

GPCRs are a major pharmaceutical target; in fact, ~50% of all current drugs target GPCRs^55^. Given that ORs are members of this pharmacologically-relevant superfamily (ORs represent the largest gene family in the genome^32^,^56^) targeting Olfr1393 may have clinical importance in the future. Because we observed an improved glucose tolerance in the *absence* of Olfr1393, from a pharmacological perspective, a key to manipulating this pathway for potential benefit may be the identification of an Olfr1393 *antagonist.* Additionally, as Sglt inhibitors are now used clinically in diabetic patients^11^,^15^,^16^, the identification of a novel regulator of Sglt1 (Olfr1393), and ultimately the physiological sources of both agonists and antagonists for Olfr1393, will improve our understanding of the Sglts and their ability to manage blood glucose.

## Materials and Methods

### Antibodies and other reagents

The polyclonal (F7425) Flag, M2 monoclonal (F1804) Flag and monoclonal acetylated tubulin (T6793) antibodies and M2 Flag beads were purchased from Sigma (St. Louis, MO). The monoclonal HA antibody (3F10) was purchased from Roche (Indianapolis, IN). The polyclonal Sglt1 and Sglt2 antibodies were generated as described previously^14^,^39^ while the monoclonal gp135 antibody was a kind gift of Dr. George Ojakian (SUNY Downstate)^26^. The β-actin antibody was purchased from Abcam (Cambridge, MA). The alexa-conjugated fluorescent secondary antibodies were purchased from Invitrogen (Carlsbad, CA) and HRP-conjugated secondary antibodies were purchased from Jackson ImmunoResearch Labs (West Grove, PA). All of the Olfr1393 ligands (cycloheptanol, cycloheptanone, cyclooctanone, cyclohexanone, 4,4 dimethylcyclohexanone, nopinone, norcamphor and 4-tertbutylcyclohexanone) were purchased from Sigma and the Dual-Luciferase Reporter Assay kit was purchased from Promega (Madison, WI). The LOPAC 1280 chemical library from Sigma was used to expand the ligand screening process. These chemicals were prepared by the Johns Hopkins ChemCore and tested at 20 μM. Any chemical that elicited a response were subsequently tested against an empty vector control to rule out Olfr1393 independent signaling events. All chemicals tested that did not elicit a Olfr1393 dependent response are listed in Supplemental Table 2.

### *In vitro* cell culture conditions

A number of different cell lines were utilized for these studies. When expressed exogenously, many ORs fail to traffic to the cell surface. HEK293T cells (co-immunoprecipitations and luciferase reporter assays) were chosen because we have previously established that Olfr1393 is properly expressed and trafficked in these cells^57^. HEK 293T cells were cultured in DMEM containing 10% FBS and supplemented with penicillin streptomycin and L-glutamine at 5% CO_2_. Polarized MDCK cells (Olfr1393 apical vs. basolateral localization) were used since they could functionally express Olfr1393, and were cultured in αMEM containing 10% FBS and supplemented with penicillin streptomycin and L-glutamine at 5% CO_2_. S3 cells are a murine proximal tubule cell line (Olfr1393 endogenous expression) and were a kind gift of Dr. Kenneth Hallows (University of Southern California). S3 cells were cultured in DMEM/F12 media containing 2% FBS and supplemented with dexamethasone, triiodothyronine, epidermal growth factor, insulin-transferrin-selenium, penicillin streptomycin and L-glutamine as previously described^21^. Another mouse proximal tubule cell line, BUMPT cells (Olfr1393 endogenous expression; kind gift of Dr. John Schwartz, Boston University) were cultured in DMEM containing 10% FBS and supplemented with penicillin streptomycin and L-glutamine on collagen-coated surfaces. As previously described^22^, cells were grown in permissive conditions in media supplemented with gamma interferon at 33 °C until ~80% confluency. Once reaching confluence, the cells were transferred to non-permissive conditions (37 °C, no gamma interferon) and allowed to fully polarize. Experimental details for all cell culture experiments are outlined below.

### Nephron segment isolation and RT-PCR

To localize Olfr1393 within the kidney, we first attempted to generate an antibody; however, our antibody attempts were not successful (GPCRs, and ORs in particular, are known to be non-antigenic^8^). Therefore, we used an RT-PCR strategy. Individual nephron segments were identified and micro-dissected from liberase-treated male kidneys as described previously^18^,^19^,^20^. During the dissection, individual segments were identified by the appearance and location within the cortex and outer medulla. Following microdissection, the segments were rinsed and RNA was extracted using the RNeasy micro kit (Qiagen; Germantown, MD). The RNA was subsequently reverse transcribed using the first strand cDNA synthesis kit (Roche Diagnostics, Meylan, France) with reverse transcriptase (RT-PCR). For segments where a sufficient amount was dissected, mock RT reactions (using water in lieu of reverse transcriptase) were performed in parallel.

### RT-PCR

Whole kidney and other tissues noted in Supplementary Table 1 were homogenized in Trizol (Life Technologies; Carlsbad, CA) and RNA was extracted by the standard phenol-chloroform protocol and treated with DNase I (Qiagen). Isolated RNA was subjected to reverse transcription (iScript Select cDNA synthesis kit; BioRad; Hercules, CA) using reverse transcriptase (RT-PCR) or water (mock RT-PCR). β-actin PCR was performed to confirm successful RNA isolation, and when positive, PCR for Olfr1393 was then performed using HotStarTaq Plus Master Mix (Qiagen) according to standard cycling protocols (35 cycles). RT and Mock RT reactions were always run simultaneously and, as expected, mock RT reactions failed to produce bands. All PCR products were sequenced to confirm identity. PCR primers used were: Olfr1393 (TAGGATGCACTGAATTGCCTTCGGG, AAACAGCTGGGCCACACACCC; 352 bp), LacZ (GAACCATCCGCTGTGGTACA, GTATCGCCAAAATCACCGCC; 606 bp) and β-actin (CGGTTCCGATGCCCTGAGGC, AGGGTGTAAAACGCAGCTCAGTAAC; 401 bp). RNA from cell lines (S3 and BUMPT cells) was isolated using the RNeasy mini kit (Qiagen). Subsequent reverse transcription and RT-PCR for Olfr1393 was completed as described above.

### Olfr1393 KO mouse

All experiments on the Olfr1393 WT and KO mice were performed in accordance with the policies and procedures of the Johns Hopkins Institutional Animal Care and Use Committee (ACUC). Experimental protocols were pre-approved by ACUC (MO16M25). The Olfr1393 KO vector and mice were designed and produced by ingenious targeting laboratory (Ronkonkoma, NY). The targeting vector was designed such that a cassette containing LacZ and an FRT-flanked Neomycin selection marker replaces the coding region of exon 1 (Olfr1393 is a single exon coding gene) (Supplementary Fig. 2A). The long homology arm (LA) extends ~5.7 kb 5′ to the site of the cassette insertion at the initiating ATG of exon 1 and the short homology arm (SA) is ~3.11 kb and extends 3′ to the site of the cassette insertion. The cassette was inserted into a C57BL/6 BAC clone (RP23: 270112) and confirmed by restriction analysis. The targeting vector was linearized and transfected by electroporation of BA1 (C57BL/6 × 129/SvEv) hybrid embryonic stem cells. After selection with G418 antibiotic, surviving clones were expanded and confirmed by both PCR analysis and Southern blotting. Positively targeted clones were injected into C57BL/6 mouse blastocysts and developed into chimera mice, which were subsequently mated to mice containing the FLP transgene to remove the Neomycin selection marker in somatic cells, and to confirm germline transmission. A further mating to wildtype mice ensured germline Neomycin deletion, leaving behind only the LacZ reporter and a 65 bp footprint of Neomycin. Heterozygous mice were bred to obtain WT and KO mice and were housed with food and water ad libitum. All studies performed on the WT and KO mice were age-matched and included both males and females.

Genotyping was performed by PCR screening of tail DNA. Briefly, tail snips were taken from 10 day old pups and lysed in lysis buffer (50 mM KCl, 2.5 mM EDTA, 50 mM Tris and 0.45% Tween-20, pH8) and 0.25 mg/ml Proteinase K overnight at 55 °C. Lysates were heated to 95 °C degrees for 15 mins and cleared at 21,000 × g for 15 mins before being used as template for a single PCR reaction (both primer sets were added to the same template) using Hotstar Plus PCR master mix (Qiagen) and standard cycling conditions (Fig. S1B). Primers used were: Common WT/KO forward - CCAAGGTGCTGGGTGAGGTCC, WT reverse - GGCACGGTGCTGGTGGTGAA, 564 bp; KO reverse – GGGGATGTGCTGCAAGGCGATT, 406 bp.

To test for expression of β-galactosidase, cryosections of WT and KO kidneys and olfactory epithelium were prepared following perfusion fixation in 4% (v/v) periodate-lysine-paraformaldehyde (PLP). β-galactosidase staining was performed using standard protocols^10^. Two β-galactosidase antibodies (rabbit polyclonal from Invitrogen and chicken polyclonal from Abcam) were additionally used to confirm expression in the OE and VNO, and to further screen other tissues.

### GTTs and ITTs

For GTTs, mice were fasted overnight for 16 hours and injected with 1 g/kg BW of glucose (Sigma) intraperitoneally. For ITTs, mice were fasted for 2 hours and injected with 0.7 U of recombinant human insulin/kg BW (Gibco). Following injection, blood was collected from a tail nick and glucose values were measured with a glucometer (Accu-Chek Nano from Roche) at 0, 15, 30, 60, 90 and 120 mins post injection.

### Other *In Vivo* Studies

Glucose and creatinine from spot urine collections were measured using the VetACE Clinical Chemistry system (Alfa Wassermann; West Caldwell, NJ). Whole blood was collected from the superficial temporal vein of conscious mice and the plasma values listed in Table 1 were obtained using the VetScan i-STAT CHEM8+ cartridge (Abaxis; Union City, CA). A drop of blood was collected by tail nick and fasted (16 h), fast/re-feed (16 h fast, 2 h re-feed) and non-fasted blood glucose was determined using a glucometer. To measure serum insulin, 30–50 μl of blood was collected from the superficial temporal vein of fasted mice and spun at 10,000 × g for 10 min to collect serum. Insulin was measured using the Rat/Mouse insulin ELISA kit (Millipore; Billerica, MA). Blood pressure was measured in conscious mice using a BP-2000 Tail Cuff Analysis System (Visitech; Apex, NC). Blood pressure was recorded daily for five consecutive days following a week of acclimation. GFR was measured in conscious, unrestrained mice by transcutaneous measurement of FITC-Sinistrin, as previously described^58^ (Mannheim Pharma & Diagnostics; Mannheim, Germany). Briefly, a small area on the back of the mice was depilated with Nair while under light isoflurane. 15 mg/100 g body weight of FITC-Sinistrin (Fresenius Kabi, Austria) was injected retroorbitally and the transcutaneous measurement device was attached to the hairless region of the back. Mice were placed back into their cage with free access to food and water for 1 hour, after which the device was removed and data downloaded. GFR was determined by the decay rate of FITC-Sinistrin using the one-compartment model at a point 15 minutes post injection. All *in vivo* data was collected from both male and female mice. The n for each study is noted in the appropriate figure legend.

### Luciferase assay and ligand screening

Olfr1393 surface expression in HEK293T cells was confirmed as described previously^57^ and ligand screening was performed using the dual-luciferase reporter assay (Promega)^31^, with all testing done in triplicate. Briefly, Flag-Olfr1393 was transfected into HEK293T cells along with constructs encoding for CREB-dependent luciferase (Firefly) and a constitutively expressed luciferase (Renilla) and RTP1S (to promote surface expression) in a black 96 well plate. 24 h after transfection, cells were incubated in stimulation media (CD293 medium with L-glutamine; Life Technologies) for 30 mins followed by 4 hours with potential odorants (diluted in the stimulation media). OR activation leads to a rise in cAMP which drives an increase in Firefly luciferase expression. Following odorant incubation, cells are rinsed with PBS, lysed and Firefly and Renilla luciferase is measured using the luciferase reporter assay kit with data collected using a FLUOstar Omega automated plate reader (BMG LabTech, Cary, NC). Firefly activity is normalized to the activity of the Renilla luciferase to control for variation in cell number and transfection efficiency and specificity of responses was determined by testing Olfr1393 and another OR in parallel.

### Cell immunofluorescence – MDCK cells

MDCK cells were grown on 12 mm Transwell polycarbonate membranes (0.4 μm pore; Sigma) and transfected with the Lucy-Flag-Olfr1393 construct that was described previously^57^ along with RTP1S^59^ (modified from RTP1L). The cleavable Lucy tag was used to promote functional expression of ORs in heterologous cells. After 24 h, the media was replaced and the cells were allowed to grow to confluency and polarize (72 h). To label the apical membrane, the top side of the Transwell membranes containing live, non-permeabilized cells was exposed to a rabbit polyclonal anti-Flag antibody (Sigma) at 4 °C. Subsequently, the cells were washed, fixed with 4% paraformaldehyde, permeabilized (0.3% Triton X-100) and exposed to a mouse gp135 antibody^26^. To detect total Flag-Olfr1393, the live cell labeling steps were skipped and the cells were immediately fixed and permeabilized and probed for Flag and gp135. The confocal images were taken with a Zeiss AxioObserver LSM700 and Zen software.

### Cell immunofluorescence – HEK293T cells

HEK293T cells were grown on 18-mm coverslips coated with poly-L-lysine and transfected with Flag-Olfr1393 or Flag-Olfr1392 with or without RTP1S. Flag-tagged OR trafficking was assessed as previously described^57^ by surface-labeling live, non-permeabilized cells at 4 °C with the polyclonal Flag antibody. Following labeling, the cells were washed, fixed with 4% paraformaldehyde, permeabilized and exposed to the mouse monoclonal (M2) Flag antibody to detect the intracellular OR population.

### Kidney immunohistochemistry

WT and KO mouse kidneys were perfusion fixed in 4% (vol/vol) PLP, and 8 μM cryosections were prepared and stained for Sglt1 or Sglt2^14^,^39^. Kidney sections were subjected to antigen retrieval in a 10 mM citrate buffer at 70 °C for 20 mins, followed by Tx-100 permeabilization (0.5% for 15 min and 2% for 30 min). After blocking in 1% BSA, sections were incubated with the polyclonal Sglt1 or Sglt2 antibody (1:500) overnight at 4 °C. The sections were then washed with PBS and Sglt staining was detected with alexa 594 secondary antibodies. Proximal tubules were labeled with LTL (Vector Labs; Burlingame, CA) and post-fixed with 4% paraformaldehyde. Confocal images were taken with a Zeiss AxioObserver LSM700 at 40X and the Z stacks were compressed with Zen software. To quantify the amount of luminal stain, random cortical fields from each kidney section were visualized and, for each LTL+/Sglt+ tubule within the field of view, the average pixel intensity was determined for (a) the luminal region and (b) whole tubule. Analysis was done using ImageJ (National Institutes of Health; Bethesda, MD). The ratio of the luminal/total Sglt stain was then determined for each tubule. The ratio from all tubules analyzed per kidney (n = 10–42 tubules) were averaged. While representative images from 2 kidneys are shown in Figs 5, 6, the quantification represents staining from 4–6 different animals/genotype.

### Co-immunoprecipitation and western blotting

HEK293T cells in 35-mm dishes were transfected for 24 h with Lucy-Flag-Olfr1393 and HA-Sglt1 either alone or together and lysed in lysis buffer containing 1% NP-40, 150 mM NaCl, 50 mM Tris and 1 mM EDTA on ice for 30 min. The lysate was cleared by centrifugation at 16,000 × g for 30 min at 4 °C and 10% of the lysate was collected in Laemmli sample buffer for later analysis. Flag-tagged ORs were then immunoprecipitated from the remaining lysate using M2 monoclonal Flag beads (Sigma). Both the immunoprecipitated fraction (B) and unbound fractions (UB) were lysed in Laemmli sample buffer and equal amounts were loaded on a gel. Proteins were transferred to a nitrocellulose membrane and immunoblotted for both Olfr1393 (using the polyclonal Flag antibody) and Sglt1 (using the monoclonal HA antibody).

Immunoblotting for Sglt1 and Sglt2 were performed on isolated total cell membranes (TCMs) from whole kidney. Sglt1 has been found in the outer stripe of the medulla (S3 segment); thus, whole kidney was selected to avoid introducing variation due to differences in dissection of the cortex and outer medulla. Briefly, whole kidneys from WT and KO mice were homogenized in 10% w/v lysis buffer (300 mM mannitol, 5 mM EGTA, 12 mM Tris pH 7.4) containing protease inhibitors and cleared at 6,000 × g for 15 min at 4 °C. To obtain the TCM, 100 μl of the kidney lysate was spun at 80,000 × g for 1 h at 4 °C and the TCM pellet was resuspended directly into Laemmli sample buffer and loaded on a gel. TCM fractions were probed for Sglt1, Sglt2^14^,^39^ and β-actin (Abcam) and the total expression of Sglt1 and Sglt2 was measured by densitometry analysis using ImageJ software and normalized to β-actin expression.

### Statistics

The student t-test was performed on all *in vivo* assays to determine differences between WT and KO mice (P < 0.05 considered significant). Olfr1393 activation by ligands was determined by a significant difference in the Firefly: Renilla ratio as compared to the non-treated control (student t-test; p < 0.05). EC_50_ values were calculated using SigmaPlot.

## Additional Information

**How to cite this article**: Shepard, B. D. *et al*. A Renal Olfactory Receptor Aids in Kidney Glucose Handling. *Sci. Rep.* **6**, 35215; doi: 10.1038/srep35215 (2016).

## Supplementary Material

Supplementary Information

## Figures and Tables

**Table 1:** Olfr1393 KO mouse phenotyping.

	WT	KO
GFR (μl/min/100 g BW)^+^	1352.2 ± 75.4	1373.0 ± 73.1
Systolic Blood Pressure* (mmHg)	115.3 ± 3.1	116.6 ± 1.6
Plasma Na^+^(mmol/L)^	144.3 ± 1.25	145.0 ± 0.66
Plasma Cl^−^ (mmol/L)^	119.3 ± 1.87	117.4 ± 0.96
Plasma iCa^+^ (mmol/L)^	1.11 ± 0.03	1.12 ± 0.01
Body Weight (g; 3-4 month old mice; n = 8)	23.51 ± 1.60	23.71 ± 1.63
Kidney Weight/Body Weight (n = 6 WT, 4 KO)	0.013 ± 0.001	0.012 ± 0.001

^+^GFR measured by transcutaneous detection of FITC-Sinistrin (n = 8)

^*^Blood pressure measured by tail cuff (n = 10 WT, 6 KO)

^plasma electrolytes measured by iStat Chem 8+ (n = 6 WT, 12 KO).
