# Supplementary material for: A Renal Olfactory Receptor Aids in Kidney Glucose Handling
Source: Sci Rep. 2016 Oct 14;6:35215. doi: 10.1038/srep35215 (PMC5064317; doi:10.1038/srep35215)
Supplement: Supplementary Information [file srep35215-s1.pdf]

Supplementary Information

A Renal Olfactory Receptor Aids in Kidney Glucose Handling

Blythe D. Shepard[1], Lydie Cheval[2], Zita Peterlin[3], Stuart Firestein[3], Hermann Koepsell[4], Alain Doucet[2], Jennifer L. Pluznick[1]

[1]Department of Physiology, Johns Hopkins University School of Medicine, Baltimore, MD 21205

[2]Sorbonne Universités, UPMC Univ Paris 06, INSERM, Université Paris Descartes, Sorbonne Paris Cité, UMR_S 1138, CNRS, ERL 8228, Centre de Recherche des Cordeliers, Paris, France.

[3]Department of Biological Sciences, Columbia University, New York, NY 10027

[4]Department of Molecular Plant Physiology and Biophysics, Julius-von-Sachs-Institute, University Wurzburg, Julius-von-Sachs-Platz 2, 97082 Wurzburg, Germany

Address all correspondences to:

Jennifer L. Pluznick, PhD
Department of Physiology
Johns Hopkins University School of Medicine
725 N. Wolfe St, WBSB 205
Baltimore, MD 21205
Ph: 410-614-4660
Email: jpluznick@jhmi.edu

Supplemental Figure 1

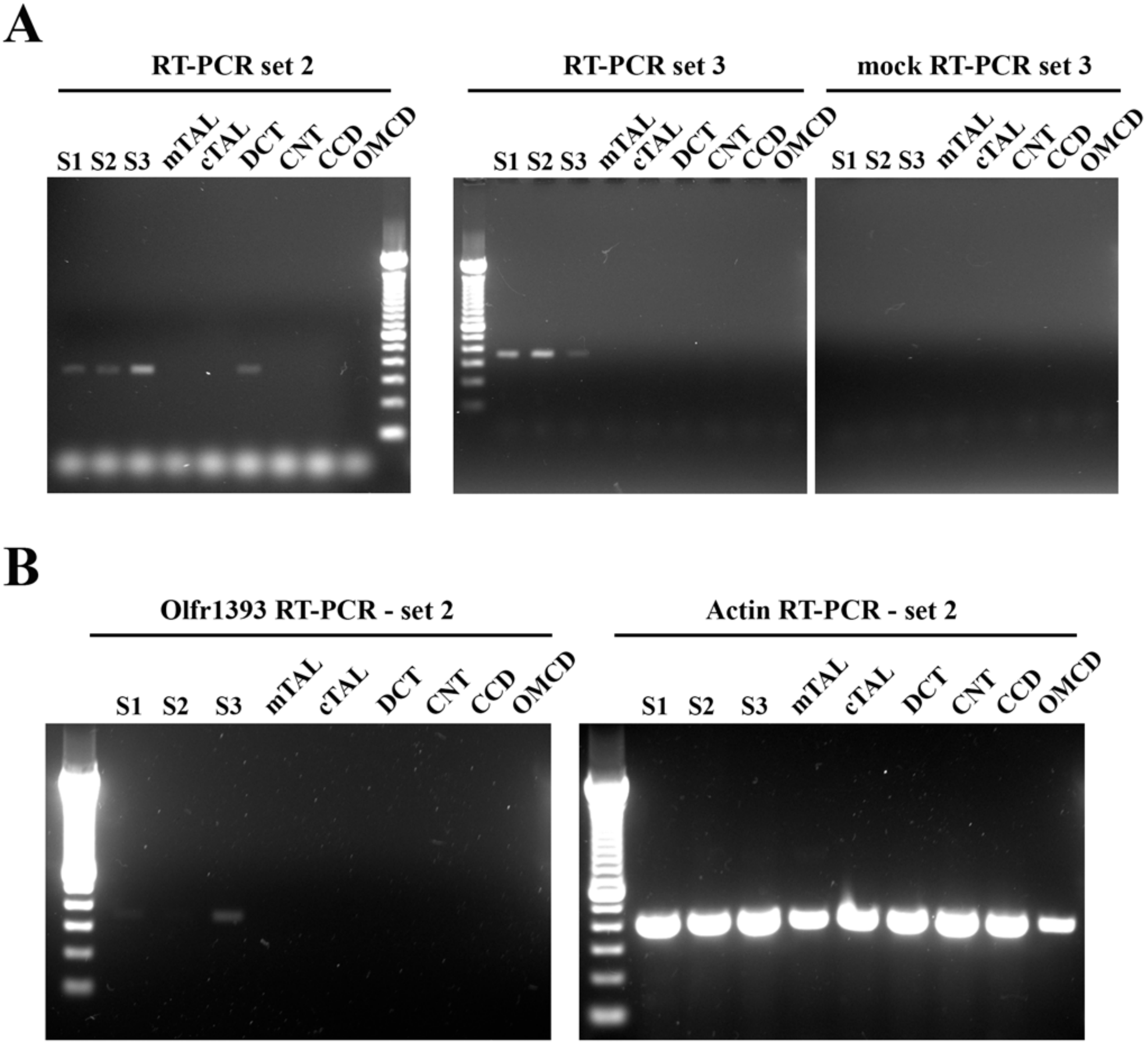

Figure S1. Olfr1393 is found in the proximal tubule by RT-PCR. A) The second and third set of hand-dissected nephron segments show that Olfr1393 is found in all three segments of the renal proximal tubule (S1, S2, S3; first set shown in Fig. 1). Where available (nephron set 3), all mock RT reactions are clean. B) Olfr1393 and actin RT-PCR performed on the second set of nephron segments and gel images were taken at the same exposure to illustrate the relative levels of expression.

Supplemental Figure 2

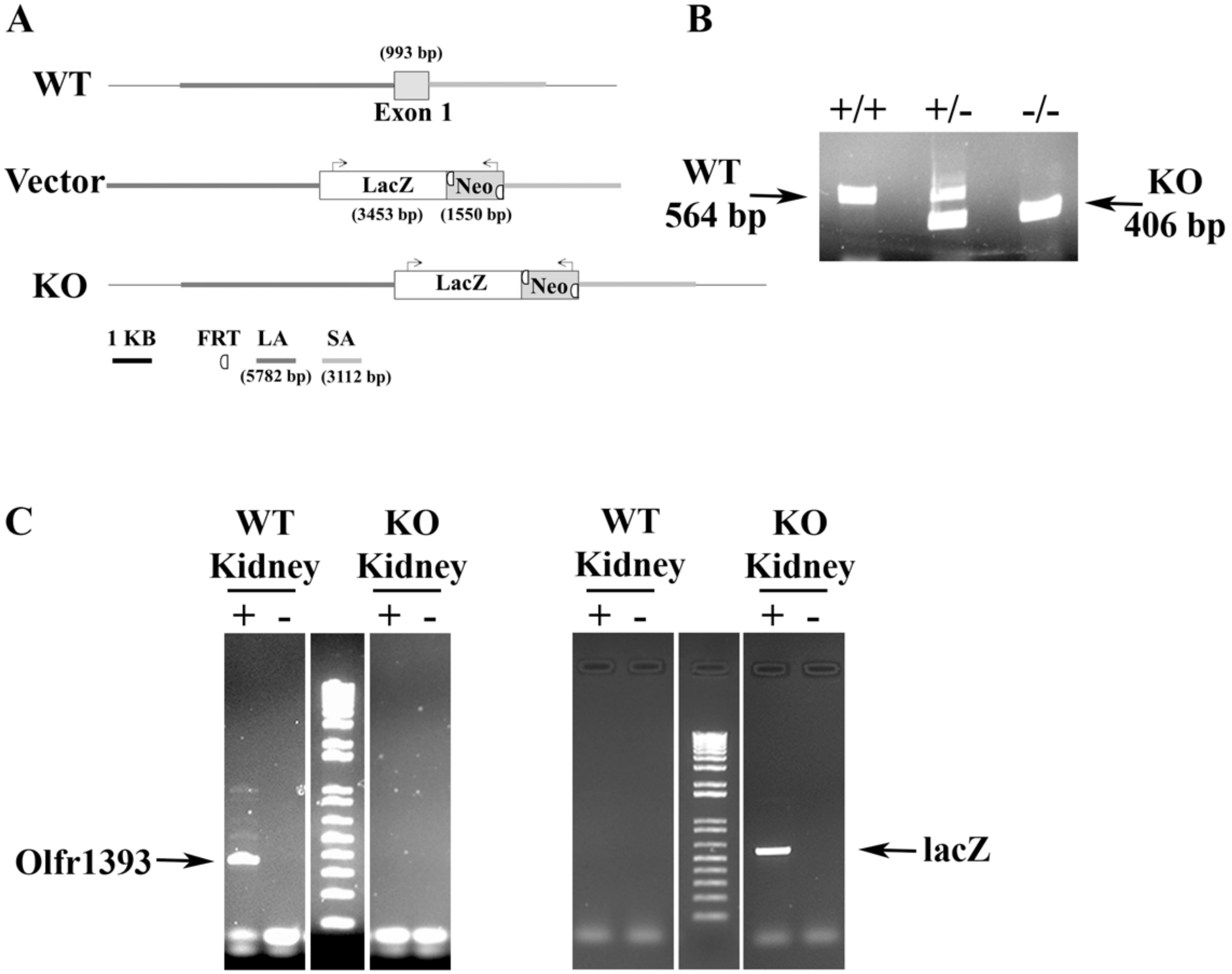

Figure S2. Generation of an Olfr1393 knockout (KO) mouse. A) A schematic representation of the KO design. To generate the KO, the entire single exon gene encoding Olfr1393 was replaced by a LacZ reporter containing a neomycin cassette. B) Genotyping from tail DNA confirms the generation of wild type (WT; +/+), heterozygous (+/-) and KO (-/-) mice. C) RNA from WT and KO kidneys were reverse-transcribed with (+) or without (-) reverse transcriptase and screened for either Olfr1393 or lacZ expression. Gene-specific primers detected Olfr1393 in the WT kidney and lacZ expression in the KO kidney.

Supplemental Figure 3

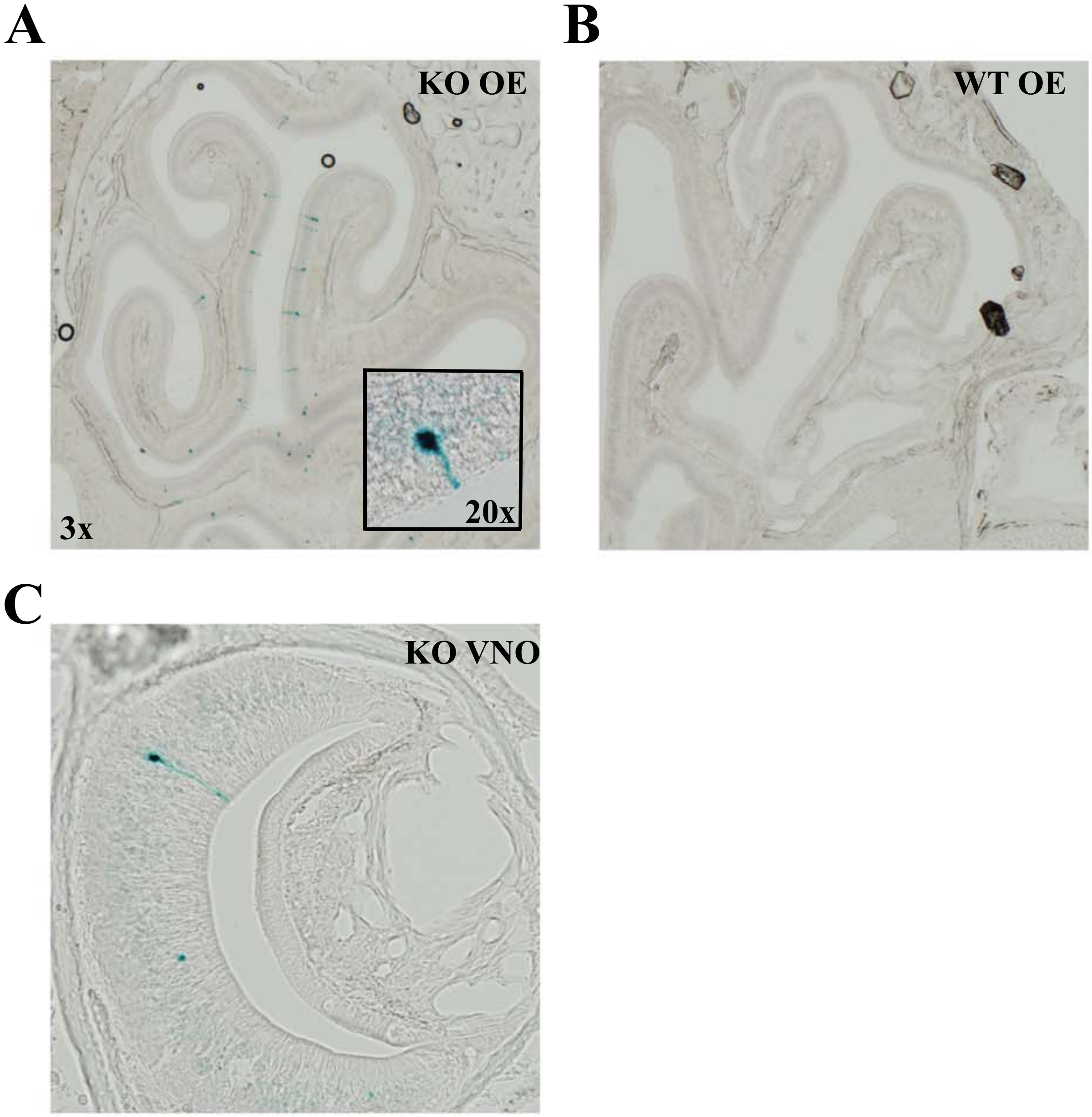

Figure S3. β-galactosidase expression is observed in the olfactory epithelium (OE) and vomeronasal organ (VNO) of Olfr1393 KO mice. WT and KO mice were perfused and cryosections were made for the OE. β-galactosidase was detected in individual neurons in the OE (A) and VNO (C) from Olfr1393 KO mice. No expression was detected in WT OE (B).

Supplemental Figure 4

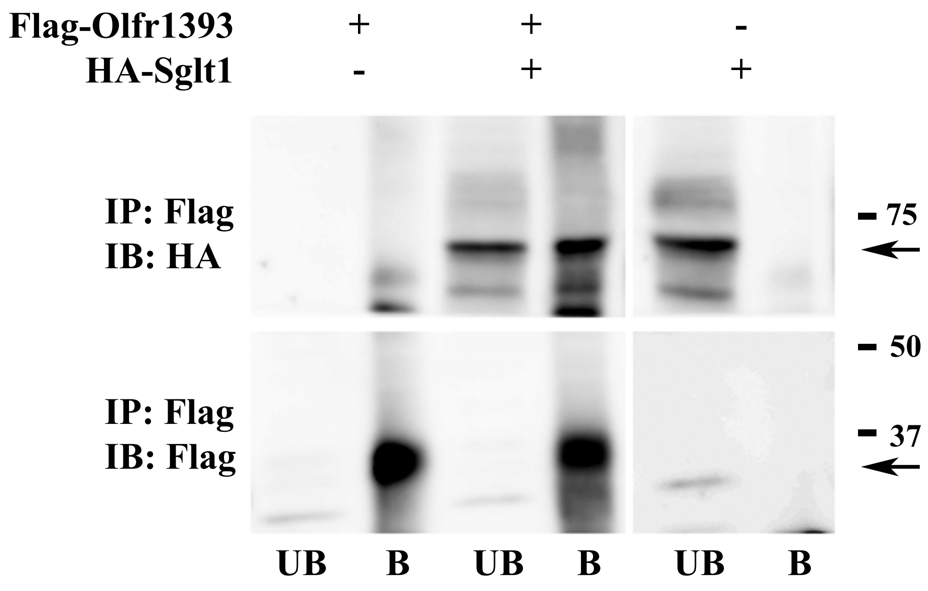

Figure S4. Olfr1393 partially co-immunoprecipitates with Sglt1. HEK293T cells expressing Flag-Olfr1393 with or without HA-Sglt1 and cells expressing HA-Sglt1 alone were lysed and immunoprecipitated for Olfr1393 using Flag-conjugated agarose beads. Both the unbound (UB) and bound (B) fractions were immunoblotted for flag to detect Olfr1393, and HA to detect Sglt1. HA-Sglt1 is found in the B fraction only when Flag-Olfr1393 is present. Arrows indicate the position of HA-Sglt1 (top gel) and Flag-Olfr1393 (bottom gel).

Supplemental Figure 5

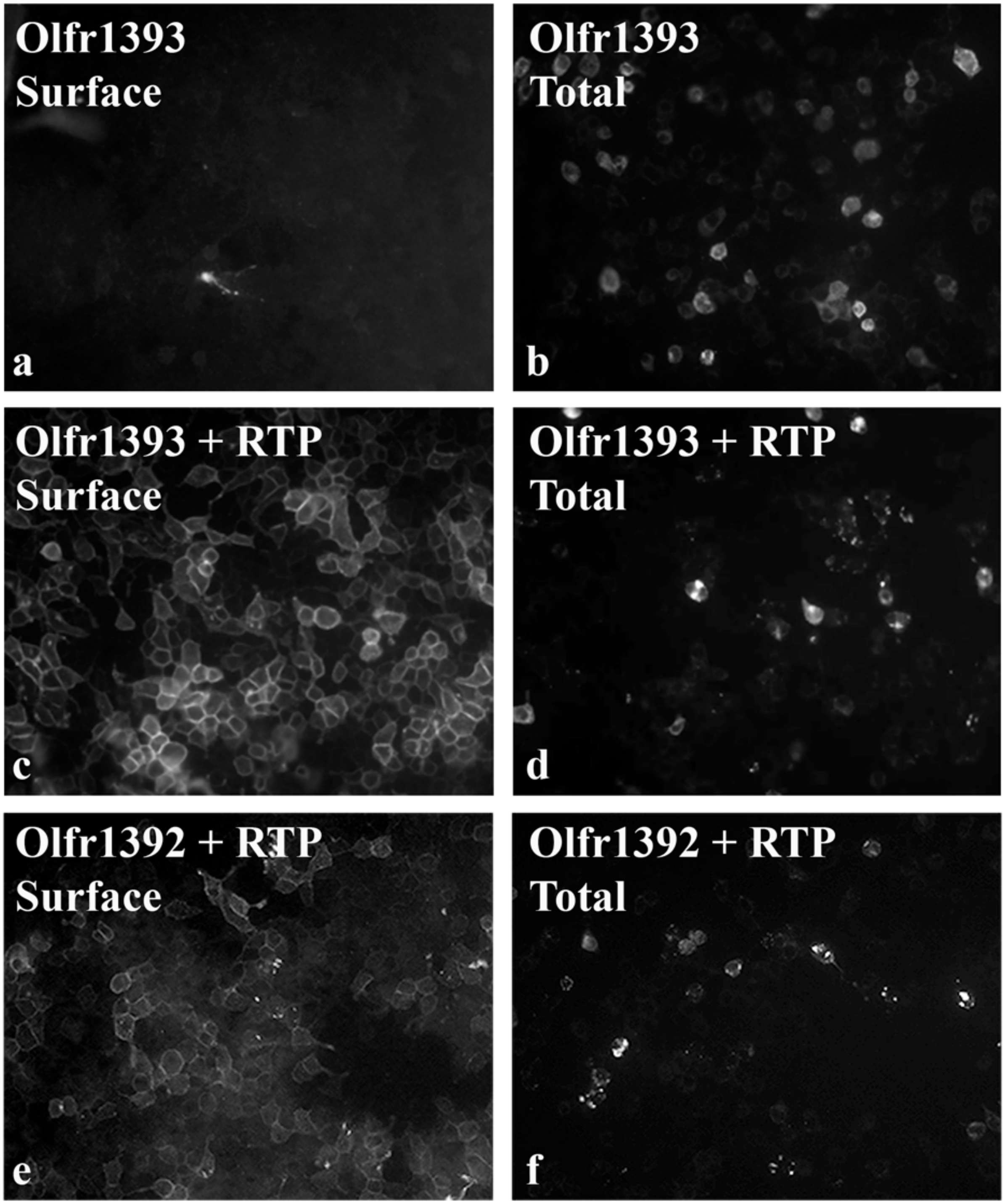

Figure S5. Olfr1393 and Olfr1392 traffic to the surface of HEK293T cells with chaperone protein RTP1S. HEK293T cells were transiently transfected with Flag-Olfr1393 with or without OR chaperone protein, RTP1S, or with Flag-Olfr1392 with RTP1S. Live, unpermeabilized cells were surface labeled with a polyclonal Flag antibody to detect Olfr1393 (a,c) or Olfr1392 (e) on the surface, and then fixed, permeabilized and stained with a monoclonal Flag antibody to detect intracellular Olfr1393 (b,d) or Olfr1392 (f). Robust surface expression is observed for both Olfr1393 and Olfr1392 in the presence of RTP1S.

Supplemental Figure 6

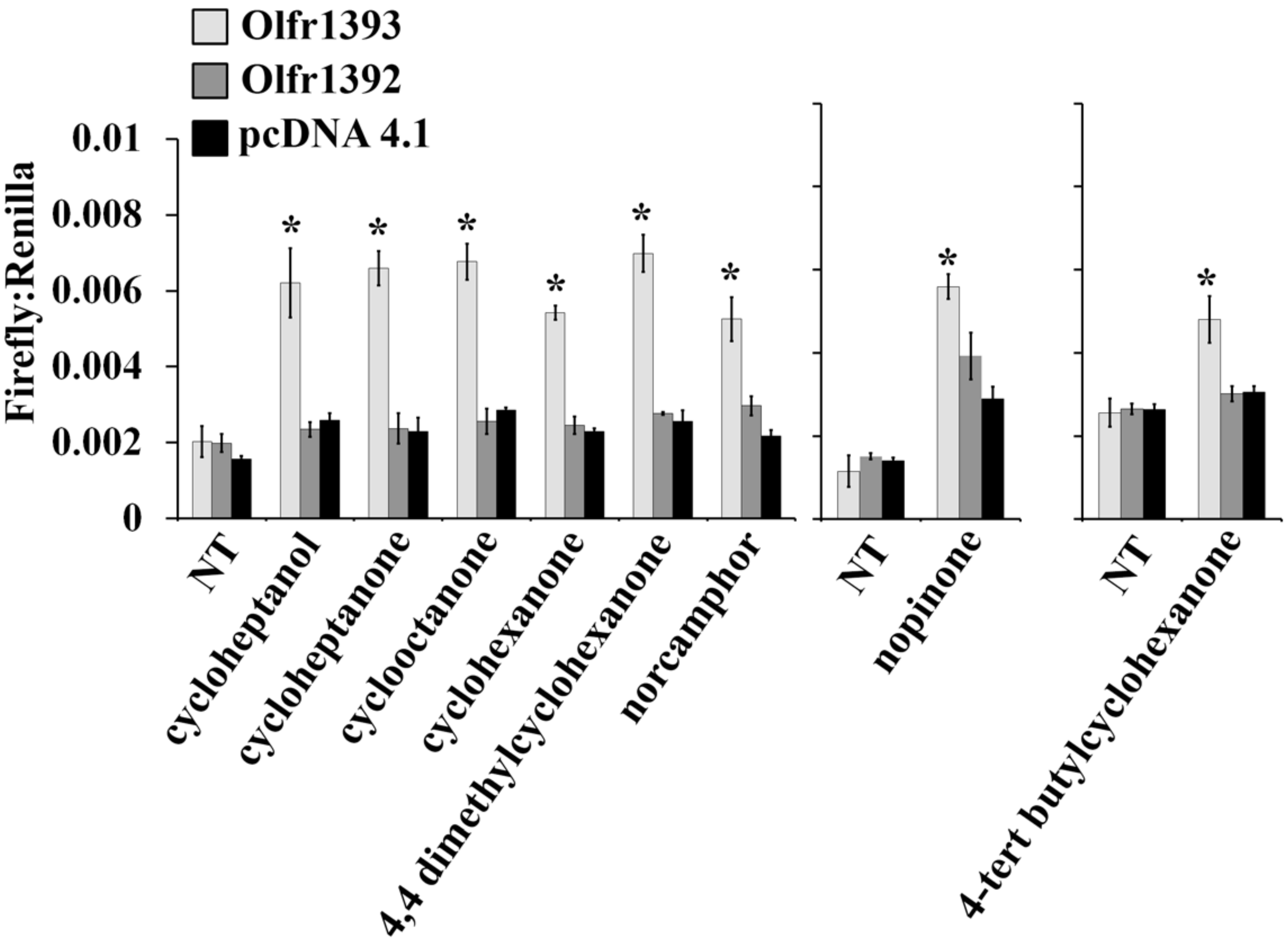

Figure S6. Olfr1393 (white bar), Olfr1392 (gray bar) and a pcDNA4.1 empty vector (black bar) were all screened against the 8 identified Olfr1393 ligands at 5mM (cycloheptanol, cycloheptanone, cyclooctanone, cyclohexanone, 4,4 dimethylcyclohexanone, norcamphor, nopinone) or 1mM (4-tert butylcyclohexanone; for this ligand, a lower dose was necessary due to cell toxicity at 5mM). In all cases Olfr1393, but not Olfr1392, responded to the ligands. Data represent ± SEM and * indicates $p \leq 0.05$ as compared to the pcDNA4.1 empty vector control. NT = non treated (stimulation media alone)

Supplemental Figure 7

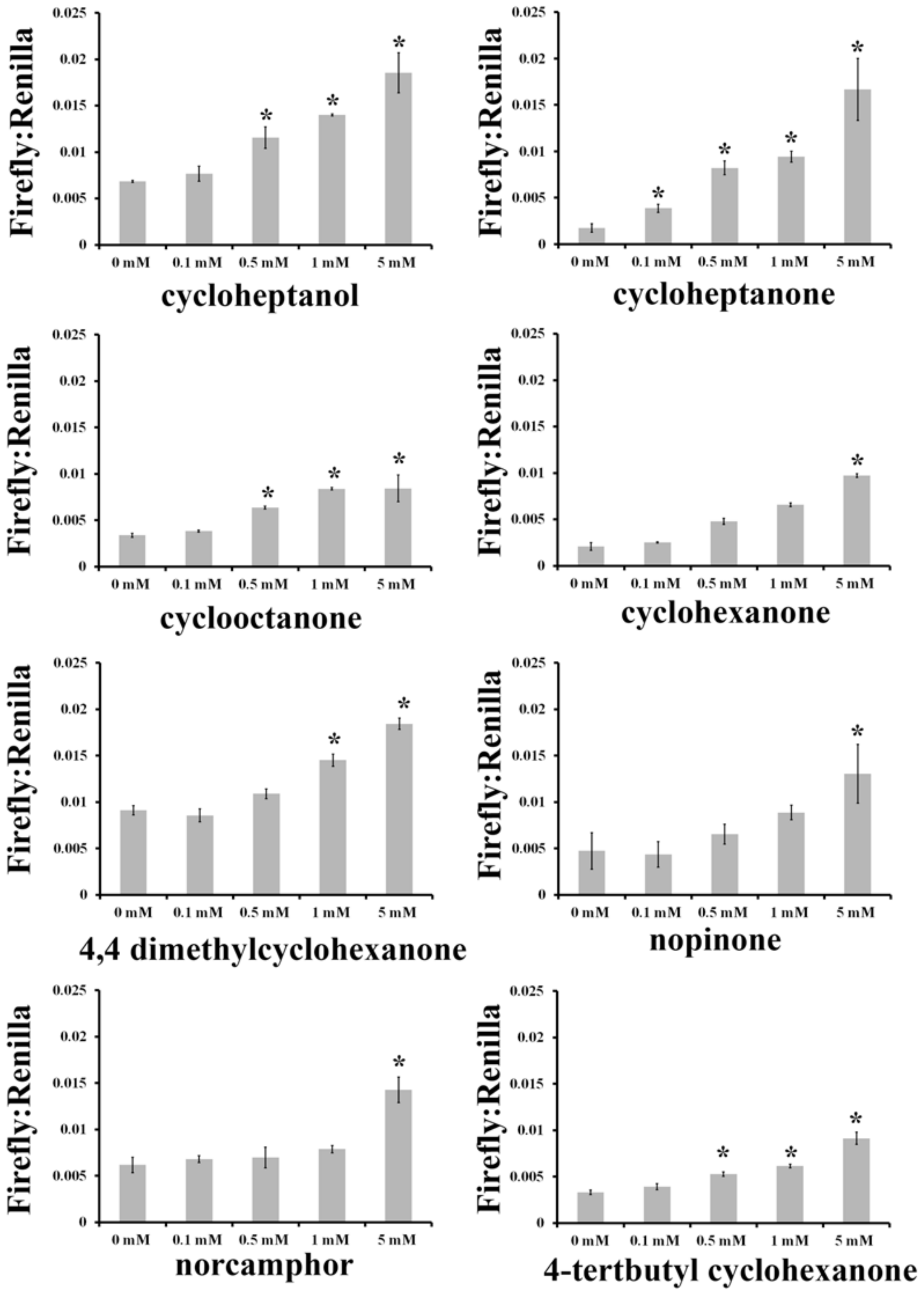

Figure S7. The complete dose responses for all 8 of the Olfr1393 ligands. Olfr1393 was screened against all of its ligands at 0, 0.1, 0.5, 1, and 5 mM and all showed a dose-dependent increase in Firefly:Renilla. The EC50 values shown in Figure 6 were calculated from these graphs. Data represent ± SEM and * indicates $p \leq 0.05$ compared to 0 mM by the student t-test.

**Always found in…**	**Frequency**
Kidney	100% (n = 8)
**Sometimes found in…**	
Brain	80% (n = 5)
Heart	67% (n = 3)
Thymus	50% (n = 4)
Lung	33% (n = 3)
Spleen	33% (n = 3)
Small Intestine	29% (n = 7)
Liver	25% (n = 8)
Adipose	20% (n = 5)
**Never found in…**	
Colon	0% (n = 4)
Pancreas	0% (n = 6)

Supplementary Table 1. Olfr1393 tissue expression

Supplemental Table 2: Olfr1393 (MOR256-24) luciferase assay testing

Chemicals listed below failed to elicit a Olfr1393 dependent response

No Response
(R)-(-)-DOI hydrochloride
(R)-(+)-WIN 55,212-2 mesylate
(-)-3-Methoxynaltrexone hydrochloride
(-)-alpha-Methylnorepinephrine
(-)Amethopterin
(-)-Bicuculline methbromide, 1(S), 9(R)
(-)-cis-(1S,2R)-U-50488 tartrate
(-)-Cotinine
(-)-Ephedrine hemisulfate
(-)-Eseroline fumarate
(-)-MK-801 hydrogen maleate
(-)-Naproxen sodium
(-)-Nicotine hydrogen tartrate salt
(-)-Perillic acid
(-)-Physostigmine
(-)-Quinpirole hydrochloride
(-)-Scopolamine hydrobromide
(-)-Scopolamine methyl bromide
(-)-Scopolamine methyl nitrate
(-)-Scopolamine,n-Butyl-, bromide
(-)-Sulpiride
(-)-Tetramisole hydrochloride
(-)-trans-(1S,2S)-U-50488 hydrochloride
(+)-Bicuculline
(+)-Bromocriptine methanesulfonate
(+)-Brompheniramine maleate
(+)-Butaclamol hydrochloride
(+)-Catechin Hydrate
(+)-Chlorpheniramine maleate
(+)-cis-Dioxolane iodide
(+)-Cyclazocine
(+)-Hydrastine
(+)-MK-801 hydrogen maleate
(+)-N-Allylnormetazocine hydrochloride
(+)-Nicotine (+)-di-p-toluoyl tartrate
(+)-Pilocarpine hydrochloride
(+)-Quisqualic acid
(+)-trans-(1R,2R)-U-50488 hydrochloride
(2S,1 S,2 S)-2-(carboxycyclopropyl)glycine
(6R)-5,6,7,8-Tetrahydro-L-biopterin hydrochloride
(Â±) trans-U-50488 methanesulfonate
(Â±)-2,3-Dichloro-alpha-methylbenzylamine hydrochloride
(Â±)-2-Amino-3-phosphonopropionic acid

(Â±)-2-Amino-4-phosphonobutyric acid
(Â±)-2-Amino-5-phosphonopentanoic acid
(Â±)-2-Amino-7-phosphonoheptanoic acid
(Â±)-3-(3,4-dihydroxyphenyl)-2-methyl-DL-alanine
(Â±)-6-Chloro-PB hydrobromide
(Â±)-7-Hydroxy-DPAT hydrobromide
(Â±)-8-Hydroxy-DPAT hydrobromide
(Â±)-alpha-Lipoic Acid
(Â±)-alpha-Methyl-4-carboxyphenylglycine
(Â±)-AMPA hydrobromide
(Â±)-AMT hydrochloride
(Â±)-Atenolol
(Â±)-Baclofen
(Â±)-Bay K 8644
(Â±)-Brompheniramine maleate
(Â±)-Butaclamol hydrochloride
(Â±)-CGP-12177A hydrochloride
(Â±)-Chloro-APB hydrobromide
(Â±)-Chlorpheniramine maleate
(Â±)-cis-Piperidine-2,3-dicarboxylic acid
(Â±)-CPP
(Â±)-DOI hydrochloride
(Â±)-gamma-Vinyl GABA
(Â±)-HA-966
(Â±)-Ibotenic acid
(Â±)-Ibuprofen
(Â±)-Methoxyverapamil hydrochloride
(Â±)-Metoprolol (+)-tartrate
(Â±)-Muscarine chloride
(Â±)-N-Allylnormetazocine hydrochloride
(Â±)-Nipecotic acid
(Â±)-Norepinephrine (+)bitartrate
(Â±)-Normetanephrine hydrochloride
(Â±)-Octoclothepin maleate
(Â±)-Octopamine hydrochloride
(Â±)-p-Aminoglutethimide
(Â±)-p-Chlorophenylalanine
(Â±)-PD 128,907 hydrochloride
(Â±)-Pindobind
(Â±)-PPHT hydrochloride
(Â±)-Propranolol hydrochloride
(Â±)-Quinpirole dihydrochloride
(Â±)-SKF 38393, N-allyl-, hydrobromide
(Â±)-SKF-38393 hydrochloride
(Â±)-Sotalol hydrochloride
(Â±)-Sulpiride
(Â±)-Synephrine

(Â±)-Taxifolin
(Â±)-Thalidomide
(Â±)-threo-1-Phenyl-2-decanoylamino-3-morpholino-1-propanol hydrochloride
(Â±)-Vanillylmandelic acid
(Â±)-Verapamil hydrochloride
(Â±)-Vesamicol hydrochloride
(E)-4-amino-2-butenoic acid
(E)-5-(2-Bromovinyl)-2 -deoxyuridine
(R,R)-cis-Diethyl tetrahydro-2,8-chrysenediol
(S)-(-)-propafenone hydrochloride
(S)-(+)-Camptothecin
(S)-3,5-Dihydroxyphenylglycine
(S)-ENBA
(S)-MAP4 hydrochloride
(S)-Propranolol hydrochloride
(Z)-Gugglesterone
1-(1-Naphthyl)piperazine hydrochloride
1-(2-Chlorophenyl)-1-(4-chlorophenyl)-2,2-dichloroethane
1-(2-Methoxyphenyl)piperazine hydrochloride
1-(3-Chlorophenyl)piperazine dihydrochloride
1-(4-Chlorobenzyl)-5-methoxy-2-methylindole-3-acetic acid
1-(4-Fluorobenzyl)-5-methoxy-2-methylindole-3-acetic acid
1-(4-Hydroxybenzyl)imidazole-2-thiol
1-(5-Isoquinolinylsulfonyl)-2-methylpiperazine dihydrochloride
1-(5-Isoquinolinylsulfonyl)-3-methylpiperazine dihydrochloride
1-(m-Chlorophenyl)-biguanide hydrochloride
1,10-Diaminodecane
1,10-Phenanthroline monohydrate
1,1-Dimethyl-4-phenyl-piperazinium iodide
1,2 cyclohexanedione
1,2 ethanedithiol
1,2,4 tri-fluorobenzene
1,3 dimethyl-2-imidazolidinone
1,3 dimethyloxybenzene
1,3,5,7 cyclooctatetraene
1,3,5-tris(4-hydroxyphenyl)-4-propyl-1H-pyrazole
1,3-Diethyl-8-phenylxanthine
1,3-Dimethyl-8-phenylxanthine
1,3-Dipropyl-7-methylxanthine
1,3-Dipropyl-8-p-sulfophenylxanthine
1,3-PBIT dihydrobromide
1,4 butanedithiol
1,4 cyclohexanedione
1,4-Dideoxy-1,4-imino-D-arabinitol
1,4-PBIT dihydrobromide
1,5 cyclooctanedione
1,5-Isoquinolinediol

1,6 hexanedithiol
1,7-Dimethylxanthine
1-[2-(Trifluoromethyl)phenyl]imidazole
10-(alpha-Diethylaminopropionyl)-phenothiazine hydrochloride
13-cis-retinoic acid
1400W dihydrochloride
16 hexadecanolide
17alpha-hydroxyprogesterone
1-Allyl-3,7-dimethyl-8-p-sulfophenylxanthine
1-Amino-1-cyclohexanecarboxylic acid hydrochloride
1-Aminobenzotriazole
1-Aminocyclopropanecarboxylic acid hydrochloride
1-benzoyl-5-methoxy-2-methylindole-3-acetic acid
1-Deoxynojirimycin hydrochloride
1-fluoropyridinium
1-heptanol
1-methyl-1-propanethiol
1-Methylhistamine dihydrochloride
1-Methylimidazole
1-octanethiol
1-octanol
1-octen-3-ol
1-octen-3-one
1-Phenyl-3-(2-thiazolyl)-2-thiourea
1-Phenylbiguanide
2 ,3 -didehydro-3 -deoxythymidine
2 ,3 -dideoxycytidine
2 aminopyridine
2 ethylfenchol
2 pentanone
2-(2-Aminoethyl)isothiourea dihydrobromide
2-(alpha-Naphthoyl)ethyltrimethylammonium iodide
2,2 -Bipyridyl
2,3 butanediol
2,3 butanedione
2,3 butanedithiol
2,3 dichloroaniline
2,3 dimethylphenol
2,3 hexanedione
2,3 pentanedione
2,3,5 trichlorophenol
2,3,6 trichlorophenol
2,3-Butanedione
2,3-Butanedione monoxime
2,3-Dimethoxy-1,4-naphthoquinone
2,4 dichloroaniline
2,4 dichlorophenol

2,4 dimethylaniline
2,4,6-trimethylaniline
2,4-Dinitrophenyl 2-fluoro-2-deoxy-beta-D-glucopyranoside
2,5 dimethylphenol
2,5 dimethylthiophene
2,5-dichlorophenol
2,6 dichlorophenol
2,6 dimethylaniline
2,6 dimethylpyrazine
2,6-Diamino-4-pyrimidinone
2,6-Difluoro-4-[2-(phenylsulfonylamino)ethylthio]phenoxyacetamide
2-butanone
2-Chloro-2-deoxy-D-glucose
2-chloro-4-fluorophenol
2-chloro-5-fluorophenol
2-chloroaniline
2-chlorophenol
2-coumaranone
2-cumaranon
2-Cyclooctyl-2-hydroxyethylamine hydrochloride
2-ethylhexanoic acid
2-fluoroaniline
2-heptanol
2-heptanone
2-hexylthiophene
2-hydroxymethylene-cyclooctanone
2-Hydroxysaclofen
2-Iodomelatonin
2-methoxyestradiol
2-methyl 4-propyl 1,3-oxathiene
2-Methyl-5-hydroxytryptamine maleate
2-methylbutyric
2-Methylthioadenosine diphosphate trisodium
2-Methylthioadenosine triphosphate tetrasodium
2-octanone
2-Phenylaminoadenosine
2-phenylethanol
3 ,4 -Dichlorobenzamil
3 -Azido-3 -deoxythymidine
3-(1H-Imidazol-4-yl)propyl di(p-fluorophenyl)methyl ether hydrochloride
3,4 dichloroaniline
3,4 hexanedione
3,4-Dichloroisocoumarin
3,4-Dihydroxyphenylacetic acid
3,5 dimethylphenol
3,5-Dinitrocatechol
3,7-Dimethyl-I-propargylxanthine

3-alpha,21-Dihydroxy-5-alpha-pregnan-20-one
3-Amino-1-propanesulfonic acid sodium
3-aminobenzamide
3-Aminopropionitrile fumarate
3-Aminopropylphosphonic acid
3-Bromo-7-nitroindazole
3-chloroaniline
3-chlorophenol
3-deazaadenosine
3-Hydroxybenzylhydrazine dihydrochloride
3-Iodo-L-tyrosine
3-Isobutyl-1-methylxanthine
3-Methoxy-4-hydroxyphenethylamine hydrochloride
3-Methoxy-morphanin hydrochloride
3-methyl-1-butanol
3-Methyl-6-(3-[trifluoromethyl]phenyl)-1,2,4-triazolo[4,3-b]pyridazine
3-Morpholinosydnonimine hydrochloride
3-Nitropropionic acid
3-n-Propylxanthine
3-octanone
3-Phenylpropargylamine hydrochloride
3-trifluoromethoxy fluorobenzene
3-trifluoromethylaniline
3-Tropanyl-3,5-dichlorobenzoate
3-Tropanyl-indole-3-carboxylate hydrochloride
3-Tropanylindole-3-carboxylate methiodide
4 chloro 3,5 dimethylphenol
4-(2-Aminoethyl)benzenesulfonyl fluoride hydrochloride
4-Amino-1,8-naphthalimide
4-Aminobenzamidine dihydrochloride
4-Aminopyridine
4-Androsten-4-ol-3,17-dione
4-Androstene-3,17-dione
4-chloro-2-methylphenol
4-chloro-3-methylphenol
4-chloroaniline
4-chlorobenzotrifluoride
4-Chloromercuribenzoic acid
4-chlorophenol
4-cycloocten-1-one
4-DAMP methiodide
4-Diphenylacetoxy-N-(2-chloroethyl)piperidine hydrochloride
4-fluoroaniline
4-Hydroxy-3-methoxyphenylacetic acid
4-Hydroxybenzhydrazide
4-Hydroxyphenethylamine hydrochloride
4-Imidazoleacrylic acid

4-Imidazolemethanol hydrochloride
4-Methoxy-3-hydroxyphenethylamine hydrochloride
4-methyl-1-phenyl-2-pentanone
4-methylcyclohexanone
4-methylcyclohexene
4-methylphenol
4-Methylpyrazole hydrochloride
4-methylvaleric acid
4-pentenoic acid
4-Phenyl-3-furoxancarbonitrile
4-tert-butylcyclohexanol
4-tert-butylcyclohexanone
5-(N,N-Dimethyl)amiloride hydrochloride
5-(N,N-hexamethylene)amiloride
5-(N-Ethyl-N-isopropyl)amiloride
5-(N-Methyl-N-isobutyl)amiloride
5,5-Dimethyl-1-pyrroline-N-oxide
5,5-Diphenylhydantoin
5,7-Dichlorokynurenic acid
5alpha-Androstane-3alpha,17beta-diol
5alpha-Pregnan-3alpha-ol-11,20-dione
5alpha-Pregnan-3alpha-ol-20-one
5-Aminovaleric acid hydrochloride
5-azacytidine
5-Bromo-2 -deoxyuridine
5-Carboxamidotryptamine maleate
5-fluoro-5 -deoxyuridine
5-Fluoroindole-2-carboxylic acid
5-Fluorouracil
5-hydroxydecanoic acid sodium
5-Hydroxyindolacetic acid
5-Hydroxy-L-tryptophan
5-Methoxy DMT oxalate
5-Nitro-2-(3-phenylpropylamino)benzoic acid
5-oxoazelaic acid
6(5H)-Phenanthridinone
6,7-ADTN hydrobromide
6,7-Dichloroquinoxaline-2,3-dione
6-Aminohexanoic acid
6-Chloromelatonin
6-Fluoronorepinephrine hydrochloride
6-Hydroxy-DL-DOPA
6-Hydroxymelatonin
6-Methoxy-1,2,3,4-tetrahydro-9H-pyrido[3,4b] indole
6-Methyl-2-(phenylethynyl)pyridine hydrochloride
6-Nitroso-1,2-benzopyrone
7,7-Dimethyl-(5Z,8Z)-eicosadienoic acid

7-Chloro-4-hydroxy-2-phenyl-1,8-naphthyridine
7-Chlorokynurenic acid
7-Cyclopentyl-5-(4-phenoxy)phenyl-7H-pyrrolo[2,3-d]pyrimidin-4-ylamine
7-Nitroindazole
8-(3-Chlorostyryl)caffeine
8-(4-Chlorophenylthio)-cAMP sodium
8-(p-Sulfophenyl)theophylline
8-Bromo-cAMP sodium
8-Bromo-cGMP sodium
8-Cyclopentyl-1,3-dimethylxanthine
8-Cyclopentyl-1,3-dipropylxanthine
8-Methoxymethyl-3-isobutyl-1-methylxanthine
9-Amino-1,2,3,4-tetrahydroacridine hydrochloride
9-cyclopentyladenine
A-315456
A-77636 hydrochloride
a-a- dimethylphenethyl acetate
AA-861
AB-MECA
AC 915 oxalate
Acetamide
Acetazolamide
acetic acid
Acetohexamide
acetone
acetophenone
acetophenone
Acetyl-beta-methylcholine chloride
Acetylsalicylic acid
Acetylthiocholine chloride
Actinonin
Acyclovir
Adenosine
Adenosine 3 ,5 -cyclic monophosphate
agarose
Agmatine sulfate
AGN 192403 hydrochloride
Agroclavine
AIDA
AL-8810
Alaproclate hydrochloride
Albuterol hemisulfate
aldosterone
Allopurinol
Alloxazine
allyl benzene
allyl phenylacetate

allyl sulfide
allylbenzene
allylphenylacetate
alpha,beta-Methylene adenosine 5 -triphosphate dilithium
alpha-Guanidinoglutaric acid
alpha-Lobeline hydrochloride
alpha-Methyl-5-hydroxytryptamine maleate
alpha-Methyl-DL-tyrosine methyl ester hydrochloride
Alprenolol hydrochloride
Altretamine
Amantadine hydrochloride
Amfonelic acid
Amifostine
Amiloride hydrochloride
Aminobenztropine
Aminoguanidine hemisulfate
Aminoguanidine hydrochloride
Aminophylline ethylenediamine
Aminopterin
Amiodarone hydrochloride
Amiprilose hydrochloride
Amitriptyline hydrochloride
Amoxapine
Amperozide hydrochloride
Amsacrine hydrochloride
Amyl Acetate
amyl hexanoate
amylhexanoate
Ancitabine hydrochloride
Androsterone
Aniracetam
anisole
Antozoline hydrochloride
Apigenin
Apomorphine hydrochloride hemihydrate
Arcaine sulfate
Arecaidine propargyl ester hydrobromide
Arecoline hydrobromide
ARL 67156 trisodium salt
Aspartic Acid
Astaxanthin
a-terpineol
ATPA
ATPO
Atropine methyl bromide
Atropine methyl nitrate
Atropine sulfate

Aurintricarboxylic acid
Azathioprine
Azelaic acid
Bay 11-7085
Beclomethasone
Benazoline oxalate
Benoxathian hydrochloride
Benserazide hydrochloride
benzaldehyde
Benzamide
Benzamidine hydrochloride
Benzamil hydrochloride
benzoic acid
Benztropine mesylate
benzyl cyanide
benzylhydrazine
Bepridil hydrochloride
Bestatin hydrochloride
beta pinene
beta-Chloro-L-alanine hydrochloride
beta-Estradiol
Betaine aldehyde chloride
Betaine hydrochloride
beta-Lapachone
Betamethasone
Betaxolol hydrochloride
Bethanechol chloride
B-HT 933 dihydrochloride
Bile Extract
B-lonone
BMY 7378 dihydrochloride
BP 897
Brefeldin A from Penicillium brefeldianum
Bretylium tosylate
BRL 15572
BRL 37344 sodium
BRL 52537 hydrochloride
BRL 54443 maleate
Bromoacetyl alprenolol menthane
Bromoacetylcholine bromide
Bromoenol lactone
BSA
BTCP hydrochloride
BU224 hydrochloride
BU99006
Budesonide
Bumetanide

Bupropion hydrochloride
Buspirone hydrochloride
butyric acid
BW 245C
BW 284c51
BW 723C86
BWB70C
Caffeic Acid
Caffeic acid phenethyl ester
Caffeine
caffeine
Calcimycin
Calmidazolium chloride
camphene
camphor
Cantharidic Acid
Cantharidin
caproic acid
Capsazepine
Captopril
Carbachol
Carbamazepine
Carbetapentane citrate
Carboplatin
Carcinine dihydrochloride
Carisoprodol
Carmustine
carvone
Carvone
CB 1954
CB34
Cefaclor
Cefazolin sodium
Cefmetazole sodium
Cefotaxime sodium
Cefsulodin sodium salt hydrate
Ceftriaxone sodium
Centrophenoxine hydrochloride
Cephalexin hydrate
Cephalosporin C zinc salt
Cephalothin sodium
Cephapirin sodium
Cephradine
Ceramide (structure altered)
CGP 20712A methanesulfonate
CGP-13501
CGP-74514A hydrochloride

CGP-7930
CGS-12066A maleate
CGS-15943
CGS-21680 hydrochloride
Chelerythrine chloride
Chelidamic acid
Chlorambucil
Chlormezanone
Chloroethylclonidine dihydrochloride
Chloro-IB-MECA
Chloroquine diphosphate
Chlorothiazide
Chlorpromazine hydrochloride
Chlorpropamide
Chlorprothixene hydrochloride
Chlorzoxazone
Choline bromide
Cilostamide
Cilostazol
Cimetidine
cinnamaldehyde
Cinnamaldehyde
Cinnarizine
Cinoxacin
Ciprofibrate
Cirazoline hydrochloride
cis-(Â±)-8-OH-PBZI hydrobromide
cis-(Z)-Flupenthixol dihydrochloride
cis-4-Aminocrotonic acid
cis-Azetidine-2,4-dicarboxylic acid
Cisplatin
Citalopram hydrobromide
Citicoline sodium
citric acid
CL 316,243
Clemastine fumarate
Clemizole hydrochloride
Clodronic acid
Clofibrate
Clomipramine hydrochloride
Clonidine hydrochloride
Clorgyline hydrochloride
Clotrimazole
Clozapine
CNQX disodium
CNS-1102
Colchicine

Cortexolone
Cortexolone maleate
Corticosterone
Cortisone
Cortisone 21-acetate
CP55940
CPCCOEt
CR 2249
CR 2945
creatinine
crotonic acid
cuminaldehyde
CV-3988
CX 546
Cyclobenzaprine hydrochloride
cyclodecanone
cycloheptane
cycloheptanol
cycloheptylamine
cyclohexanecarboxylic
cyclohexanol
cyclohexyl acetate
cyclohexyl isocyanate
cyclohexylamine
cyclooctane
cyclopentanol
Cyclophosphamide monohydrate
Cyclosporin A
Cyclothiazide
Cyproheptadine hydrochloride
Cyproterone acetate
Cystamine dihydrochloride
Cysteamine hydrochloride
Cytosine-1-beta-D-arabinofuranoside hydrochloride
D(-)-2-Amino-7-phosphonoheptanoic acid
D-609 potassium
Daidzein
Danazol
Dantrolene sodium
Daphnetin
DBO-83
DCEBIO
D-Cycloserine
Debrisoquin sulfate
Decamethonium dibromide
decanoic acid
Dehydroisoandrosterone 3-sulfate sodium

Demeclocycline hydrochloride
deoxycholic acid
Dephostatin
Dequalinium analog, C-14 linker
Dequalinium dichloride
Desipramine hydrochloride
Dextromethorphan hydrobromide monohydrate
Dextrorphan D-tartrate
Diacylglycerol kinase inhibitor I
Diacylglycerol Kinase Inhibitor II
Diazoxide
dibenzyl ether
Diclofenac sodium
Dicyclomine hydrochloride
dicyclopentadiene
Diethylenetriaminepentaacetic acid
Dihydrexidine hydrochloride
Dihydro-beta-erythroidine hydrobromide
Dihydrocapsaicin
dihydrocarvone
Dihydroergocristine methanesulfonate
Dihydroergotamine methanesulfonate
Dihydrokainic acid
Dihydroouabain
Dilazep hydrochloride
Diltiazem hydrochloride
Diphenhydramine hydrochloride
Diphenyleneiodonium chloride
Dipropyldopamine hydrobromide
Dipyridamole
Disopyramide
Disopyramide phosphate
DL-alpha-Difluoromethylornithine hydrochloride
DL-alpha-Methyl-p-tyrosine
DL-Buthionine-[S,R]-sulfoximine
DL-Cycloserine
DL-erythro-Dihydrosphingosine
DL-Homatropine hydrobromide
DL-p-Chlorophenylalanine methyl ester hydrochloride
DL-Stearoylcarnitine chloride
DL-Thiorphan
DL-threo-beta-hydroxyaspartic acid
DM 235
DNQX
Dobutamine hydrochloride
Domperidone
Dopamine hydrochloride

Doxazosin mesylate
Doxepin hydrochloride
Doxycycline hydrochloride
Doxylamine succinate
DPMA
D-ribofuranosylbenzimidazole
Droperidol
D-Serine
DSP-4 hydrochloride
Dubinidine
d-valerolactone
E-64
Ebselen
E-decalactone
Edrophonium chloride
Efaroxan hydrochloride
Ellipticine
Emetine dihydrochloride hydrate
Emodin
Endothall
Enoximone
Epibestatin hydrochloride
epinephrine
Ergocristine
erythro-9-(2-Hydroxy-3-nonyl)adenine hydrochloride
estradiol
Estrone
ET-18-OCH3
Etazolate hydrochloride
Ethosuximide
ethyl benzoate
Ethylene glycol-bis(2-aminoethylether)-N,N,N ,N -tetraacetic acid
Etodolac
Etoposide
eugenol
Famotidine
Farnesylthiosalicylic acid
FBS
Felbamate
Felodipine
Fenofibrate
Fenoldopam bromide
Fenspiride hydrochloride
Fexofenadine hydrochloride
Fiduxosin hydrochloride
Flecainide acetate
Flumazenil

Flunarizine dihydrochloride
Fluoxetine hydrochloride
Fluphenazine dihydrochloride
Flupirtine maleate
Fluspirilene
Flutamide
Fluvoxamine maleate
Foliosidine
Formate
Formoterol
FPL 64176
FSCPX
Furafylline
Furegrelate sodium
Furosemide
Fusaric acid
Fusidic acid sodium
GABA
Gabaculine hydrochloride
Gabapentin
galactose
Gallamine triethiodide
gamma-Acetylinic GABA
gamma-D-Glutamylaminomethylsulfonic acid
Ganciclovir
GBR-12909 dihydrochloride
GBR-12935 dihydrochloride
Genistein
geraniol
Glibenclamide
Glipizide
glucosamine
glucose
glucose
glucuronic acid
Glutamic Acid
glycine
GR 113808
GR 125487 sulfamate salt
GR 127935 hydrochloride
GR 4661
GR-89696 fumarate
Guanabenz acetate
Guanfacine hydrochloride
Guanidinoethyl disulfide dihydrobromide
Guanidinyl-naltrindole di-trifluoroacetate
Guvacine hydrochloride

GW1929
GW2974
GW5074
GW7647
GW9662
GYKI 52466 hydrochloride
GYKI 52895
H-7 dihydrochloride
H-8 dihydrochloride
H-89
H-9 dihydrochloride
HA-100
HA-1004 hydrochloride
Haloperidol
Harmane
Hemicholinium-3
Heptanal
heptanoic acid
Hexahydro-sila-difenidol hydrochloride, p-fluoro analog
Hexamethonium bromide
Hexamethonium dichloride
hexamethyleneimine
Hexanoic acid
Hispidin
Histamine dihydrochloride
Histamine, R(-)-alpha-methyl-, dihydrochloride
Hydralazine hydrochloride
Hydrochlorothiazide
Hydrocortisone
hydrocortisone (cortisol)
Hydrocortisone 21-hemisuccinate sodium
Hydroquinone
Hydroxylamine hydrochloride
Hydroxytacrine maleate
Hydroxyurea
Hypotaurine
Ibudilast
IC 261
ICI 118,551 hydrochloride
ICI 204,448 hydrochloride
Icilin
Idarubicin
Idazoxan hydrochloride
IEM-1460
Ifenprodil tartrate
IIK7
Imazodan

Imetit dihydrobromide
IMID-4F hydrochloride
Imidazole-4-acetic acid hydrochloride
Imiloxan hydrochloride
Imipramine hydrochloride
Indatraline hydrochloride
Indirubin-3 -oxime
indole
Indomethacin
Indomethacin morpholinylamide
Iodoacetamide
Iofetamine hydrochloride
I-OMe-Tyrphostin AG 538
Ipratropium bromide
Iproniazid phosphate
isobutyrate
isocaproic acid
Isoguvacine hydrochloride
Isoliquiritigenin
Isonipecotic acid
isophorone
isosafrole
isovaleric acid
Isoxanthopterin
Ivermectin
JL-18
JWH-015
K 185
Kainic acid
Karakoline
Kenpaullone
Ketanserin tartrate
Ketoconazole
Ketoprofen
Ketorolac tris salt
Ketotifen fumarate
Kynurenic acid
L(-)-Norepinephrine bitartrate
L-162,313
L-165,041
L-2-aminoadipic acid
L-3,4-Dihydroxyphenylalanine
L-3,4-Dihydroxyphenylalanine methyl ester hydrochloride
L-368,899
L-655,240
L-655,708
L-687,384 hydrochloride

L-701,324
L-703,606 oxalate
L-732,138
L-733,060 hydrochloride
L-741,626
L-745,870 hydrochloride
L-750,667 trihydrochloride
L-765,314
Labetalol hydrochloride
lactic acid
L-allylglycine
L-alpha-Methyl DOPA
L-alpha-Methyl-p-tyrosine
Lamotrigine
Lansoprazole
L-Arginine
L-Aspartic acid
L-azetidine-2-carboxylic acid
L-Buthionine-sulfoximine
L-Canavanine sulfate
L-Cycloserine
L-Cysteinesulfinic Acid
LE 300
Leflunomide
leucine
Levallorphan tartrate
LFM-A13
L-Glutamic acid hydrochloride
L-Glutamic acid, N-phthaloyl-
L-glutamine
L-Histidine hydrochloride
L-Hyoscyamine
Lidocaine hydrochloride
Lidocaine N-ethyl bromide quaternary salt
Lidocaine N-methyl hydrochloride
limonene
limonene (-)
linalool
Linopirdine
Lithium Chloride
L-Leucinethiol, oxidized dihydrochloride
L-Methionine sulfoximine
L-N5-(1-Iminoethyl)ornithine hydrochloride
L-N6-(1-Iminoethyl)lysine hydrochloride
Lomefloxacin hydrochloride
Lonidamine
Loperamide hydrochloride

Loratadine
Lorglumide sodium
Loxapine succinate
loxoprofen
L-Tryptophan
Luteolin
LY-278,584 maleate
LY-294,002 hydrochloride
LY-310,762 hydrochloride
LY-367,265
LY-53,857 maleate
lyral
lyral
M1745 Sigma Amino Acids
maleic anhydride
malonic acid
maltose
Maprotiline hydrochloride
McN-A-343
m-cresol
MDL 105,519
MDL 26,630 trihydrochloride
MDL 28170
Me-3,4-dephostatin
Mecamylamine hydrochloride
Meclofenamic acid sodium
Melatonin
Meloxicam sodium
Melphalan
Memantine hydrochloride
Mesulergine hydrochloride
Metaphit methanesulfonate
Metaproterenol hemisulfate
Metergoline
Methapyrilene hydrochloride
Methiothepin mesylate
Methoctramine tetrahydrochloride
Methotrexate
Methoxamine hydrochloride
Methyl 6,7-dimethoxy-4-ethyl-beta-carboline-3-carboxylate
Methyl beta-carboline-3-carboxylate
methyl isoeugenol
methyl nonanoate
Methylcarbamylcholine chloride
methylcyclohexane
Methylergonovine maleate
methylmalonic acid

Methysergide maleate
Metoclopramide hydrochloride
Metolazone
Metrazoline oxalate
Mevastatin
Mexiletene hydrochloride
MG 624
MHPG piperazine
MHPG sulfate potassium
Mianserin hydrochloride
Mibefradil dihydrochloride
Mifepristone
Milrinone
Minocycline hydrochloride
Minoxidil
m-Iodobenzylguanidine hemisulfate
Mitoxantrone
Mizoribine
MJ33
MK-886
MK-912
ML-7
ML-9
Molsidomine
Monastrol
Morin
Moxisylyte hydrochloride
Moxonidine hydrochloride
MRS 1523
MRS 1754
MRS 1845
MRS 2159
MRS 2179
m-toluidine
Muscimol hydrobromide
musk ketone
Myricetin
N-(2-[4-(4-Chlorophenyl)piperazin-1-yl]ethyl)-3-methoxybenzamide
N-(3,3-Diphenylpropyl)glycinamide
N-(4-Amino-2-chlorophenyl)phthalimide
N-(4-Aminobutyl)-5-chloro-2-naphthalenesulfonamide hydrochloride
N-(p-Isothiocyanatophenethyl)spiperone hydrochloride
N,N,N ,N -Tetramethylazodicarboxamide
N,N,N-trimethyl-1-(4-trans-stilbenoxy)-2-propylammonium iodide
N,N-Dihexyl-2-(4-fluorophenyl)indole-3-acetamide
N,N-Dipropyl-5-carboxamidotryptamine maleate
N^G,N^G-Dimethylarginine hydrochloride

N6-2-(4-Aminophenyl)ethyladenosine
N6-Cyclopentyl-9-methyladenine
N6-Methyladenosine
N6-Phenyladenosine
Na pyruvate
N-Acetyl-5-hydroxytryptamine
N-Acetyldopamine monohydrate
N-Acetyl-L-Cysteine
N-Acetylprocainamide hydrochloride
N-Acetyltryptamine
NaCl
NADPH tetrasodium
Naftopidil dihydrochloride
Nalbuphine hydrochloride
Nalidixic acid sodium
Naloxonazine dihydrochloride
Naloxone benzoylhydrazone
Naloxone hydrochloride
Naltrexone hydrochloride
Naltriben methanesulfonate
Naltrindole hydrochloride
NAN-190 hydrobromide
Naphazoline hydrochloride
Na-p-Tosyl-L-lysine chloromethyl ketone hydrochloride
N-arachidonylglycine
NBI 27914
NBQX disodium
N-Bromoacetamide
NCS-356
NCS-382
Neostigmine bromide
nerolidol
N-Ethylmaleimide
NF 023
NF449 octasodium salt
NG-Hydroxy-L-arginine acetate
NG-Monomethyl-L-arginine acetate
NG-Nitro-L-arginine
NG-Nitro-L-arginine methyl ester hydrochloride
Nialamide
Nicardipine hydrochloride
Niclosamide
Nifedipine
Niflumic acid
Nilutamide
Nimesulide
Nimodipine

Nimustine hydrochloride
Nisoxetine hydrochloride
Nitrendipine
N-Methyl-1-deoxynojirimycin
N-Methyl-beta-carboline-3-carboxamide
N-Methyl-D-aspartic acid
N-Methyldopamine hydrochloride
NO-711 hydrochloride
Nocodazole
N-Oleoyldopamine
N-Oleoylethanolamine
N-omega-Methyl-5-hydroxytryptamine oxalate salt
Nomifensine maleate
Nonanal
nonanoic
nor-Binaltorphimine dihydrochloride
Norcantharidin
Nordihydroguaiaretic acid from Larrea divaricata (creosote bush)
Nortriptyline hydrochloride
Noscapine hydrchloride
N-Phenylanthranilic acid
N-p-Tosyl-L-phenylalanine chloromethyl ketone
NS 2028
NS 521 oxalate
NS-1619
NSC 95397
N-Succinyl-L-proline
N-Vanillylnonanamide
Nylidrin hydrochloride
O-(Carboxymethyl)hydroxylamine hemihydrochloride
O6-benzylguanine
o-cresol
octahydro-2H-chromen-2-one
octanal
octanethiol
Octanoic acid
ODQ
Ofloxacin
Oleic Acid
Oligomycin A
Olomoucine
O-Methylserotonin hydrochloride
O-Phospho-L-serine
Orphenadrine hydrochloride
o-toluidine
Ouabain
OXA-22 iodide

Oxaprozin
Oxatomide
Oxiracetam
Oxolinic acid
Oxotremorine methiodide
Oxotremorine sesquifumarate salt
Oxybutynin Chloride
Oxymetazoline hydrochloride
P1,P4-Di(adenosine-5)tetraphosphate triammonium
Palmitoyl-DL-Carnitine chloride
Palmitoylethanolamide
p-Aminoclonidine hydrochloride
Pancuronium bromide
Papaverine hydrochloride
PAPP
Pargyline hydrochloride
Paromomycin sulfate
Parthenolide
p-Benzoquinone
PD 168,077 maleate
PD 404,182
PD 98,059
Pempidine tartrate
pentachloroaniline
pentachlorophenol
pentafluorobenzene
Pentamidine isethionate
Pentanal
Pentanal
Pentolinium di[L(+)-tartrate]
Pentoxifylline
Pentylenetetrazole
pentylfuran
Pergolide methanesulfonate
Perphenazine
p-Fluoro-L-phenylalanine
Phaclofen
Phenamil methanesulfonate
Phenelzine sulfate
Pheniramine maleate
phenol
Phenoxybenzamine hydrochloride
Phentolamine mesylate
Phenylbenzene-omega-phosphono-alpha-amino acid
Phenylbutazone
Phenylephrine hydrochloride
phenylethyl isobutyrate

Phenytoin sodium
Phloretin
Phosphomycin disodium
Phosphonoacetic acid
Phosphoramidon disodium
Phthalamoyl-L-glutamic acid trisodium
Piceatannol
Picotamide
Picrotoxin
Pilocarpine nitrate
pimelic acid
Pimozide
Pinacidil
Pindolol
p-Iodoclonidine hydrochloride
piperidine
Piperidine-4-sulphonic acid
Piracetam
Pirenperone
Pirenzepine dihydrochloride
Pirfenidone
Piribedil maleate
Piroxicam
PK 11195
p-MPPF dihydrochloride
p-MPPI hydrochloride
Podophyllotoxin
potassium nitrate
PPADS
PPNDS tetrasodium
Praziquantel
Prazosin hydrochloride
PRE-084
Pregnenolone sulfate sodium
Prilocaine hydrochloride
Primidone
Procainamide hydrochloride
Procaine hydrochloride
Prochlorperazine dimaleate
Progesterone
Proglumide
Promazine hydrochloride
Promethazine hydrochloride
Propafenone hydrochloride
Propantheline bromide
Propentofylline
Propionylpromazine hydrochloride

Propofol
Protoporphyrin IX disodium
Protriptyline hydrochloride
Purvalanol A
Putrescine dihydrochloride
Pyrazinecarboxamide
Pyridostigmine bromide
Pyrilamine maleate
Pyrocatechol
pyruvaldehyde
Quazinone
Quercetin dihydrate
Quinacrine dihydrochloride
Quinelorane dihydrochloride
Quinidine sulfate
Quinine sulfate
Quinolinic acid
Quipazine dimaleate
Quipazine, 6-nitro-, maleate
Quipazine, N-methyl-, dimaleate
R(-)-2,10,11-Trihydroxyaporphine hybrobromide
R(-)-2,10,11-Trihydroxy-N-propylnoraporphine hydrobromide
R(-)-Apocodeine hydrochloride
R(-)-Denopamine
R-(-)-Deprenyl hydrochloride
R-(-)-Desmethyldeprenyl hydrochloride
R-(-)-Fluoxetine hydrochloride
R(-)-Me5
R(-)-N-Allylnorapomorphine hydrobromide
R(-)-Propylnorapomorphine hydrochloride
R(-)-SCH-12679 maleate
R(+)-3PPP hydrochloride
R(+)-6-Bromo-APB hydrobromide
R-(+)-7-Hydroxy-DPAT hydrobromide
R-(+)-8-Hydroxy-DPAT hydrobromide
R(+)-Atenolol
R(+)-Butylindazone
R(+)-IAA-94
R(+)-Lisuride hydrogen maleate
R(+)-SCH-23390 hydrochloride
R(+)-Terguride
R(+)-UH-301 hydrochloride
R7131 Sigma Amino Acids
rac-2-Ethoxy-3-hexadecanamido-1-propylphosphocholine
rac-2-Ethoxy-3-octadecanamido-1-propylphosphocholine
Raloxifene hydrochloride
Ranitidine hydrochloride

Ranolazine dihydrochloride
Rauwolscine hydrochloride
Reactive Blue 2
Reserpine
Resveratrol
Retinoic acid
Retinoic acid p-hydroxyanilide
REV 5901
Ribavirin
Rilmenidine hemifumarate
Riluzole
Risperidone
Ritanserin
Ritodrine hydrochloride
Ro 04-6790 dihydrochloride
Ro 16-6491 hydrochloride
Ro 20-1724
Ro 25-6981 hydrochloride
Ro 41-0960
Ro 41-1049 hydrochloride
Ro 8-4304
Rolipram
Ropinirole hydrochloride
Roscovitine
Rotenone
Rottlerin
Rp-cAMPS triethylamine
Rutaecarpine
Ruthenium red
RX 821002 hydrochloride
S(-)-3PPP hydrochloride
S(-)-Atenolol
S-(-)-Carbidopa
S(-)-DS 121 hydrochloride
S-(-)-Eticlopride hydrochloride
S(-)-IBZM
S-(-)-Lisuride
S(-)-p-Bromotetramisole oxalate
S(-)-Pindolol
S(-)-Timolol maleate
S(-)-UH-301 hydrochloride
S(-)-Willardiine
S-(+)-Fluoxetine hydrochloride
S(+)-Ibuprofen
S(+)-PD 128,907 hydrochloride
S(+)-Raclopride L-tartrate
S-(4-Nitrobenzyl)-6-thioguanosine

S-(4-Nitrobenzyl)-6-thioinosine
S-(p-Azidophenacyl)glutathione
S-5-Iodowillardiine
Salmeterol
Sandoz 58-035
Sanguinarine chloride
SB 200646 hydrochloride
SB 202190
SB 203186
SB 204070 hydrochloride
SB 204741
SB 205384
SB 206553 hydrochloride
SB 216763
SB 218795
SB 222200
SB 224289 hydrochloride
SB 228357
SB 269970 hydrochloride
SB 415286
SB-366791
SC 19220
SC-560
SCH-202676 hydrobromide
SCH-28080
SDZ-205,557 hydrochloride
Se-(methyl)selenocysteine hydrochloride
Seglitide
Semicarbazide hydrochloride
Sepiapterin
Serotonin hydrochloride
S-Ethylisothiourea hydrobromide
SIB 1757
SIB 1893
SKF 75670 hydrobromide
SKF 83565 hydrobromide
SKF 83959 hydrobromide
SKF 86466
SKF 89626
SKF 89976A hydrochloride
SKF 91488 dihydrochloride
SKF 94836
SKF 95282 dimaleate
SKF 96365
SKF 97541 hydrochloride
SKF-525A hydrochloride
S-Methylisothiourea hemisulfate

S-Methyl-L-thiocitrulline acetate
SNC80
S-Nitrosoglutathione
S-Nitroso-N-acetylpenicillamine
Sobuzoxane
sodium acetate
sodium citrate
sodium formate
sodium malonate
Sodium nitroprusside dihydrate
sodium oxalate
Sodium Oxamate
sodium oxylate
sodium phosphate
sodium propionate
sodium pyruvate
sodium succinate
Sodium Taurocholate
SP600125
Spermidine trihydrochloride
Spermine tetrahydrochloride
Sphingosine
Spiperone hydrochloride
Spironolactone
Spiroxatrine
SQ 22536
SR 2640
SR 57227A
SR 59230A oxalate
SR-95531
SU 4312
SU 5416
SU 6656
succinic acid
Succinylcholine chloride
sucrose
Sulfaphenazole
Sulindac
Sulindac sulfone
Suramin hexasodium
T-0156
T-1032
Tamoxifen citrate
Taurine
Taxol
TCPOBOP
Telenzepine dihydrochloride

Terazosin hydrochloride	
Terfenadine	
testosterone	
Tetracaine hydrochloride	
Tetradecylthioacetic acid	
Tetraethylammonium chloride	
Tetraethylthiuram disulfide	
Tetrahydrozoline hydrochloride	
Tetraisopropyl pyrophosphoramide	
Tetramisole hydrochloride	
TFPI hydrochloride	
Thapsigargin	
Theobromine	
Theophylline	
Thiocitrulline	
Thiolactomycin	
Thio-NADP sodium	
Thioperamide maleate	
Thioridazine hydrochloride	
Thiothixene hydrochloride	
THIP hydrochloride	
Tiapride hydrochloride	
TMB-8 hydrochloride	
Tolazamide	
Tolbutamide	
Tomoxetine	
TPMPA	
Tracazolate	
Tranilast	
trans-(Â±)-ACPD	
trans,trans 2,4 heptadienal	
trans-2-octen-1-ol	
trans-2-octenal	
trans-Azetidine-2,4-dicarboxylic acid	
trans-Dehydroandrosterone	
Tranylcypromine hydrochloride	
Trazodone hydrochloride	
trehalose	
Triamcinolone	
Triamterene	
Trifluoperazine dihydrochloride	
Trifluperidol hydrochloride	
Triflupromazine hydrochloride	
Trihexyphenidyl hydrochloride	
Trimethoprim	bolded = ne
trimethylamine N-oxide	
Trimipramine maleate	

Triprolidine hydrochloride
Tropicamide
Tryptamine hydrochloride
TTNPB
Tulobuterol hydrochloride
Tyrphostin 1
Tyrphostin 23
Tyrphostin 25
Tyrphostin 47
Tyrphostin 51
Tyrphostin A9
Tyrphostin AG 112
Tyrphostin AG 126
Tyrphostin AG 1478
Tyrphostin AG 34
Tyrphostin AG 490
Tyrphostin AG 494
Tyrphostin AG 527
Tyrphostin AG 528
Tyrphostin AG 537
Tyrphostin AG 538
Tyrphostin AG 555
Tyrphostin AG 698
Tyrphostin AG 808
Tyrphostin AG 835
Tyrphostin AG 879
U0126
U-101958 maleate
U-62066
U-69593
U-73122
U-74389G maleate
U-75302
U-83836 dihydrochloride
U-99194A maleate
UK 14,304
Urapidil hydrochloride
Urapidil, 5-Methyl-
urea
uric acid
Uridine 5 -diphosphate sodium
valeric acid
valine
valproic acid
Valproic acid sodium
Vancomycin hydrochloride from Streptomyces orientalis
Vanillic acid diethylamide

VER-3323 hemifumarate salt
Vinblastine sulfate salt
Vincristine sulfate
Vinpocetine
vitamins
VUF 5574
W-7 hydrochloride
WAY-100635 maleate
WB 64
WB-4101 hydrochloride
WIN 62,577
Wortmannin from Penicillium funiculosum
w-penta decalactone
Xamoterol hemifumarate
xanthine
Xanthine amine congener
XK469
Xylazine hydrochloride
Xylometazoline hydrochloride
Y-27632 dihydrochloride
y-butyrolactone
YC-1
Yohimbine hydrochloride
YS-035 hydrochloride
Zaprinast
Zardaverine
Zimelidine dihydrochloride
Z-L-Phe chloromethyl ketone
ZM 39923 hydrochloride
Zonisamide sodium
Zopiclone
